# The position of visual word forms in the anatomical and representational space of visual categories in occipitotemporal cortex

**DOI:** 10.1162/imag_a_00196

**Published:** 2024-06-25

**Authors:** Ineke Pillet, Begüm Cerrahoğlu, Roxane Victoria Philips, Serge Dumoulin, Hans Op de Beeck

**Affiliations:** Department of Brain and Cognition, Leuven Brain Institute, KU Leuven, Leuven, Belgium; 2LPN (Laboratoire Lorrain de Psychologie et Neurosciences de la Dynamique des Comportements), Université de Lorraine, France; Department of Cognitive and Behavioral Sciences, University of Luxembourg, Esch-sur-Alzette, Luxembourg; Spinoza Centre for Neuroimaging, Amsterdam, Netherlands; Computational Cognitive Neuroscience and Neuroimaging, Netherlands Institute for Neuroscience, Amsterdam, Netherlands; Experimental and Applied Psychology, Vrije University Amsterdam, Amsterdam, Netherlands; Experimental Psychology, Helmholtz Institute, Utrecht University, Utrecht, Netherlands

**Keywords:** visual word form area, occipitotemporal cortex, 7T fMRI

## Abstract

Recent reviews emphasized the need for investigating the complexity of multiplesubareas of word selectivity and how this relates to selectivity for othervisual categories, at the individual level at a high spatial resolution (withoutnormalization or smoothing). To investigate this, both on the brain surface andin the representational space of the occipitotemporal cortex, we presented 19participants with images of 20 different categories during 7T fMRI. Thesecategories included several word-like conditions, and in addition cover many ofthe dimensions that have been suggested to define object space, such as animacyand real-world size. In the left hemisphere, we found three subareas of thevisual word form area (VWFA) and one extra subarea around the pFusface-selective area. We also observed several areas of selectivity to hands thatcould consistently guide the localization of word and face areas. No clearpredictive anatomical landmarks were found. Results of the right hemisphere wereless clear, in part due to weaker word selectivity. In the representationalspace, word selectivity stood out from other categories. It had multipleneighboring categories at a similar distance (e.g., faces, bodies, hands, cars),so no special relationship was found with, for example, faces. These resultsenable a consistent and reliable way to locate subareas of word selectivity andmay inspire future research into words in the representational space of theoccipitotemporal cortex.

## 1 Introduction

The occipitotemporal cortex (OTC) supports visual object recognition (Barton et al., 2002;Gaillard et al., 2006;Grill-Spector etal., 2000;Moutoussis & Zeki,2002). It contains local areas selective to certain visual categories,like faces (Kanwisher et al., 1997;Tsao et al., 2006), bodies (Downing et al., 2001;Peelen & Downing, 2005), hands (Bracci et al., 2010;Bratch et al.,2018), tools (Bracci et al., 2012;Peelen et al., 2013), scenes (Aguirre et al., 1998;Epstein & Kanwisher, 1998), word forms (Baker et al., 2007;Cohen et al., 2000,^2002^), and numerals (Abboud et al.,2015,Shum et al., 2013). Inaddition, response patterns distributed throughout the OTC represent othercategories, as detected using multi-voxel pattern analysis (MVPA) (Haxby et al., 2001;Kriegeskorte, 2008).

Within OTC, category selectivity is organized according to dimensions on whichcategories vary. For example, in ventral OTC, areas responsive to animate categoriesare located more laterally than those to inanimate categories (Grill-Spector & Weiner, 2014). This dimension alsoorganizes representational spaces as detected through MVPA (for a classic example,seeKriegeskorte, 2008). Other examples oforganizing dimensions are real-world size (Konkle& Oliva, 2012) and body topography (Orlov et al., 2010). More low/mid-level visual dimensionslike retinotopy also influence OTC’s functional organization (Malach et al., 2002). For example, both face and wordareas prefer visual information provided by the fovea (Grill-Spector et al., 2017;Hasson et al., 2002;Leet al., 2017;Lerma-Usabiaga et al.,2021;Rauschecker et al.,2012).

The location of some category-selective areas can be predicted from certainanatomical landmarks and/or from the location of other areas of selectivity. Forexample,Weiner et al. (2014)found that theanterior tip of the mid-fusiform sulcus, that separates the fusiform gyrus into alateral and medial part, predicts the location of fusiform face area 2 (FFA-2), alsocalled mFus (middle fusiform face area). As another example,Weiner and Grill-Spector (2010)observed that face- andlimb-selective areas alternate in a consistent pattern, enabling a reliable way todefine face areas (mFus, pFus, and IOG) based on their spatial relation tolimb-selective areas (along OTS and ITG) (Weiner& Grill-Spector, 2010). This alternation appeared more consistentacross subjects in the right hemisphere (Weiner& Grill-Spector, 2010). The authors suggested that this might beinfluenced by the presence of word-selective areas in the left hemisphere (Weiner & Grill-Spector, 2010), althoughthey could not test this due to the absence of a word form category in theirstudy.

Weiner’s and Grill-Spector’s (2010) suggestion, that the face-limb areaalternation is not consistent in the left hemisphere due to words, is grounded inresearch that proposes a competition for cortical territory in OTC between faces andwords during development. This research is based on the neuronal recycling theory(Dehaene & Cohen, 2007;Dehaene et al., 2010). This theory states thatneural circuits for similar evolutionary older skills (face processing) arerepurposed for recently invented skills (written language) (Dehaene & Cohen, 2007). The specifics of how thisrecycling unfolds is a topic of debate. Some studies indicated a destructivecompetition (Dehaene et al., 2010), butothers indicated no competition but rather a fine-tuning of visual objectrecognition (Hervais-Adelman et al., 2019). Arecent study adds another category on top of faces and words to the mix:Nordt et al. (2021)found increased selectivityfor faces and words and decreased selectivity for limbs during learning to read andwrite. This suggests an interplay between destructive competition and visualfine-tuning. This challenges the specificity of the proposed competition betweenfaces and words.

The word selectivity in ventral OTC is often called the visual word form area (VWFA),extending from the posterior occipitotemporal sulcus (OTS) to about the midpoint ofthe fusiform gyrus (Yeatman et al., 2021).Two recent studies aimed to subdivide this large region based on functionaldifferences.Lerma-Usabiaga et al. (2018)applied two types of contrasts (perceptual and lexical), revealing that the anteriorregion (mOTS) was more sensitive to lexical characteristics of the stimuli and wasstructurally more connected to language areas, compared to the posterior region(pOTS). The authors stated that the mOTS corresponds to the central VWFA (asdescribed inCohen & Dehaene, 2004;Cohen et al., 2002;Dehaene et al., 2002;Vogel et al., 2012) and pOTS to posterior VWFA (as described inBen-Shachar et al., 2007;Vogel et al., 2012).White et al. (2019)investigated selective spatial attention of the VWFAand concluded that VWFA-1 could in parallel process multiple words, whereas VWFA-2processed only one word at a time (after an integration of hemifields). VWFA-1 andVWFA-2 correspond to pOTS and mOTS.Yeatman et al.(2021)proposed that VWFA-1 lies next to FFA-1 and VWFA-2 next to FFA-2and in between those, lies a body/limb-selective area (see alsoGrill-Spector & Weiner, 2014). These studiesdivided the large VWFA into two subareas based on functional properties rather thanclear anatomical boundaries.

Several researchers recently emphasized the need for high anatomical precision at thelevel of the individual brain (without spatial normalization or smoothing) whenstudying the location of the VWFA and possible subareas (Caffarra et al., 2021;Yeatman et al., 2021). Such single-subject precision impacts accuratedescriptions of spatial organization: the VWFA may seem like one large region due toa lack of such precision (Caffarra et al.,2021). With a higher precision, the VWFA may be subdivided based onanatomical boundaries instead of (only) based on functional differences. Thus, inour study, we mapped word selectivity (obtained through a general linear model) onthe anatomy of the ventral brain surface of each of the 19 subjects that werescanned with 7T fMRI. This allowed a higher spatial resolution, investigating eachsubject separately, and without applying normalization or smoothing preprocessingsteps. This type of study will allow for consistency in the defining subareas of theVWFA and facilitates studies investigating functional distinctions between thesesubareas (Caffarra et al., 2021;Yeatman et al., 2021).

In addition to the words category, our study included various potentially relevantcategories. This allows word selectivity to be related to other categoryselectivity. Such a relation may be expected based on findings from, for example,Weiner and Grill-Spector (2010)who foundconsistent spatial relations between face- and limb-selective areas (but they didnot include word-selective areas). Findings from the neural recycling theory suggestthat categories of faces, hands, and bodies are relevant for the functionalneuroanatomy of word selectivity. Building onYeatman et al. (2021), we also expected bodies and hands (a type oflimb) to be relevant because they might lie between two subregions of the VWFA.While one recent study byBoring et al. (2021)did explore faces compared to words at a high spatial resolution in the individualsubject space, our study goes further by including several other relevant visualcategories (faces, hands, bodies, and other character categories: fake script andnumbers) on the word selectivity map and relating them to each other. In addition,we ran a split-half analysis to replicate category selectivity and to gain insightsinto the response profile of these selective areas. Uncovering a spatial relationbetween word and other category-selective areas can enhance the reliability offuture studies in locating category-selective areas.

Our study included many categories that vary on the multiple dimensions that organizeobject space (such as animacy, object size, and retinotopy), and can thus contributeto investigating word selectivity in the representational space, by using MVPA.Based on the competition between words and faces proposed by the neural recyclingtheories, our study investigates if a special relation between these categories alsoexists in the representational space. To construct this space, our study included atotal of 20 categories varying on several dimensions like animacy and objectsize.

## 2 Methods

### 2.1 Subjects

Nineteen subjects participated in the study (mean age: 30.1 ± 6.8 (23-45),sex: 11 males, 8 females). Sixteen subjects were right-handed, and three wereleft-handed (subject 2, 7, and 17). All subjects had normal or corrected tonormal visual acuity. Every subject gave informed consent. All procedures wereapproved by the ethics committee of Vrije Universiteit Amsterdam and adhered tothe guidelines of the Declaration of Helsinki.

### 2.2 Stimuli

Stimuli (500 x 500 pixels, 4.7 degrees visual angle) were presented on a32ʹʹ LCD screen (69.8 × 39.3 cm, 120 Hz) designed to use inan MRI environment (BOLDscreen, Cambridge Research Systems, UK). The screenresolution was 1920 × 1080 pixels. The screen was positioned at the endof the bore and viewed through a mirror (distance from screen: 220 cm) mountedon the head coil. All stimuli were presented with MATLAB (MathWorks, Inc.) andthe Psychophysics Toolbox Version 3 (Brainard,1997;Kleiner et al., 2007;Pelli, 1997). Stimuli were presentedat a semi-random position around the middle of the screen (center of stimulusmaximum 33 pixels/0.32 degrees visual angle away) in every trial, to avoidlow-level visual confounds (same motivation as for applying the SHINE toolbox tothe stimuli, described below) and to make the one-back task less trivial. Afixation dot was always present in the middle of the screen, presented over thestimuli.

The experiment was designed in a way that it could be interesting for differentresearch purposes. We chose 20 conditions (19 categories + 1 scrambledcontrol condition) to have a rich and varied set according to several criteria.The set included both natural and artificial objects, known and unknown shapes(cubies and smoothies, seeOp de Beeck et al.,2006), animate and inanimate shapes, objects with a differentreal-world size, objects differing in how they are used and whether they are atool or not. The categories could be grouped together in several ways, dependingon the exact purpose of the research, according to dimensions such as these.Within each category, different viewpoints/angles on the objects were includedand there was sufficient variability in the identity of the stimuli. Thefollowing is an alphabetical list of all the categories, for each an example isalso depicted inFigure 1: bodies,buildings, cars, cats, chairs, cubies, faces, fake script, fish, flowers,hammers, hands, musical instruments, numbers, scissors, scrambled, smoothies,trees, vegetables, words. For the purposes of this study, certain categorieswere of particular interest as described in detail in the introduction (althoughall of them were of interest to construct the representational space of OTC):words, faces, other characters than words (numbers and fake script), otherhuman-related and thus animate categories than faces (bodies and hands), and atypical object category to also include an inanimate category in the set, thatcould be used as a comparison and reference landmark (chairs). We did not choosethe buildings category as this would activate too medial parts of ventral OTC toserve as a control for the categories of interest. InFigure 1, the character group is framed in purple, therelevant animate categories in red, and the reference inanimate category ingreen.

**Fig. 1.**
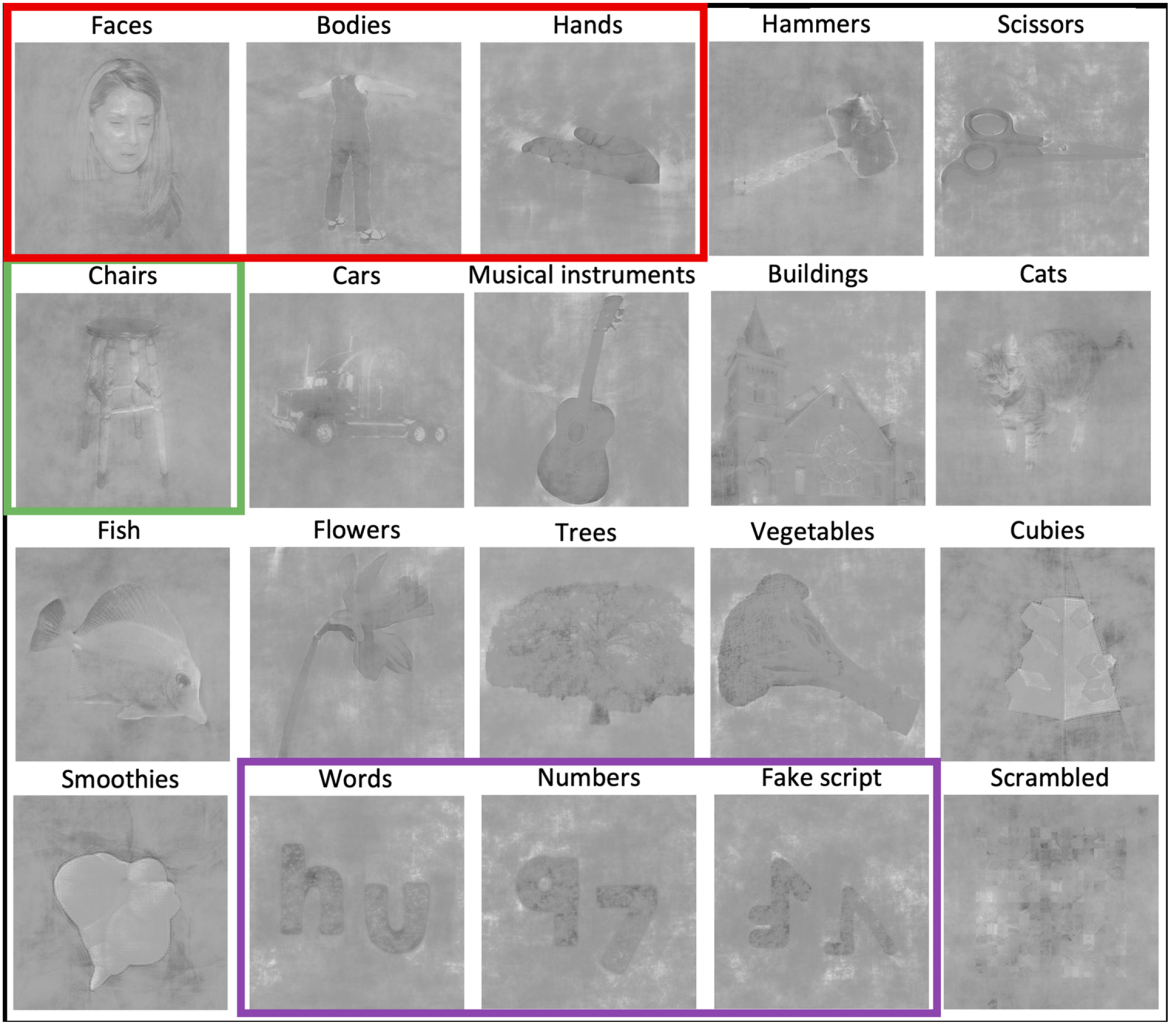
For each of the 20 categories, an example stimulus is shown. The categorythe stimulus belongs to is indicated on top of the image. The charactercategories are framed in purple, the relevant animate (human-related)categories in red, and the typical object category used as a referencein green.

In the creation of the stimuli, we attempted to minimize the role of low- andmid-level features in the differences between categories. For eight categories(bodies, buildings, cars, cats, chairs, faces, fish, hammers), stimuli wereprovided byCohen et al. (2017). Theyensured high within-category variability by, for example, images of items indifferent angles, in different positions (Cohenet al., 2017). As described above, we also followed these guidelinesin the creation of the other categories’ images. The following categories(partially) consist of images from the Bank of Standardized Stimuli (BOSS):musical instruments, flowers, vegetables, scissors (Brodeur et al., 2010). To add more images to thesecategories, and to create stimuli for other categories (hammers, hands, trees),we created stimuli ourselves by using freely available images on theinternet.

The words, the fake script, and the numbers categories consisted of two, three,or four letters/characters with a variable aspect ratio achieved by an ascendingor descending angle of the string. They were presented in a bold font filledwith a random dotted pattern. Half of the stimuli of each category werepresented onto a white background and the other half on a black background,ensuring comparability in retinotopic envelope with other categories. The wordslacked a semantic meaning. The words all contained at least 1 vowel (except oneof the 30 stimuli) and were all pronounceable. For simplicity, we refer to thiscategory as words rather than letter strings or pseudo-words. The fake scriptstimuli were created using a combination of two fonts to ensure the letterswould take on a varied shape (e.g., short, long, blocked, curvy).

The control category (scrambled images) was created using the randblock(rgb)function, that is available online for MATLAB (Mathworks, Inc.), on all otherimages in the stimulus set. From all these scrambled images, we chose 30 images(including at least one from each of the other 19 conditions) where the middleof the image contained more variability than the borders as this resembles thegeneral way in which the other categories’ images were organized.

For the unknown objects we used the cubies and smoothies stimuli described in thestudy byOp de Beeck et al. (2006). Bothobjects could vary on four different shape-dimensions. On each of thedimensions, the object had a value from 0 to 5. We chose 16 shapes for bothcubies and smoothies that took an extreme position in this 4D-space (e.g., 0505,0005) and four more that had a center position within this space (e.g., 2222,2323). Ten of those 20 stimuli were duplicated before any further processinghappened. All 30 stimuli were then flipped with a random angle, making sure theduplicates were angled differently than their original version, to end up with30 unique stimuli. During creation, the objects’ positions on the greybackground were ensured to not always be in the center.

Several conditions required extra control to match low and mid-level featuresbetween categories, on top of ensuring within-condition variability inviewpoints and identity. Hammers and scissors are often elongated in shape. Toavoid retinotopic confounds, we translated and rotated exemplars, includeddifferent viewpoints, and selected the scissors open instead of closed. Wecontrolled for the specific shape of trees by using different kinds of trees, bysizing them smaller/larger, and by not always having them appear in the middlepart of the background but also on the upper/lower left/right part. We replacedthe images of the buildings category that contained trees with other images ofbuildings that did not contain trees, as trees is supposed to be a separatecategory. The bodies’ images were also adapted so they would have novisible hands, as this is a category on its own.

In the end, 30 stimuli were available for each category. All stimuli were thenmatched for average luminance, contrast, and spectral energy by the SHINEtoolbox (Willenbockel et al., 2010),inspired by the study ofCohen et al.(2017). Afterward, the identity of each stimulus was ensured to be atleast moderately clear/visible to viewers. We conducted several additionalchecks: a mean greyvalue image, a standard deviation greyvalue image, and anedge image were created for each category and then compared between categoriesto ensure they did not differ.

All images, and in addition all data (including raw data of all participants inthe surface space) relevant to the analyses presented in this study, areavailable in the following GIN repository:https://doi.org/10.12751/g-node.96eqnl(Pillet et al., 2024).

### 2.3 Experimental design

Subjects were explained the task before entering the scanner. They were asked tofocus on the fixation dot in the center of the screen. A dual task was used. Inthe first task, a one-back task, subjects pressed a button with the instructedhand (which hand changed every subject) when an image was an identical copy ofthe image of the previous trial (seeFig.2B, top row). Thus, if the same object was presented, but rotated orangled differently, they did not press the button. In the second task, subjectspressed a button, with their opposite hand, as soon as possible when a change incategory occurred (seeFig. 2B, bottomrow). This ensured subjects were processing category information and not justmore low-level features to perform the first task. To familiarize subjects withthe categories, an example stimulus from each category was shown after taskexplanation. Overall, participants showed a high performance in the categorytask, with a hit rate of 85% and a false alarm rate of 1%. The one-bask task wasmore difficult, with a hit rate of 65% and a false alarm rate of 10%. The lattercan be explained by the fast pace of the stimuli in a block (one every 0.67seconds).

**Fig. 2.**
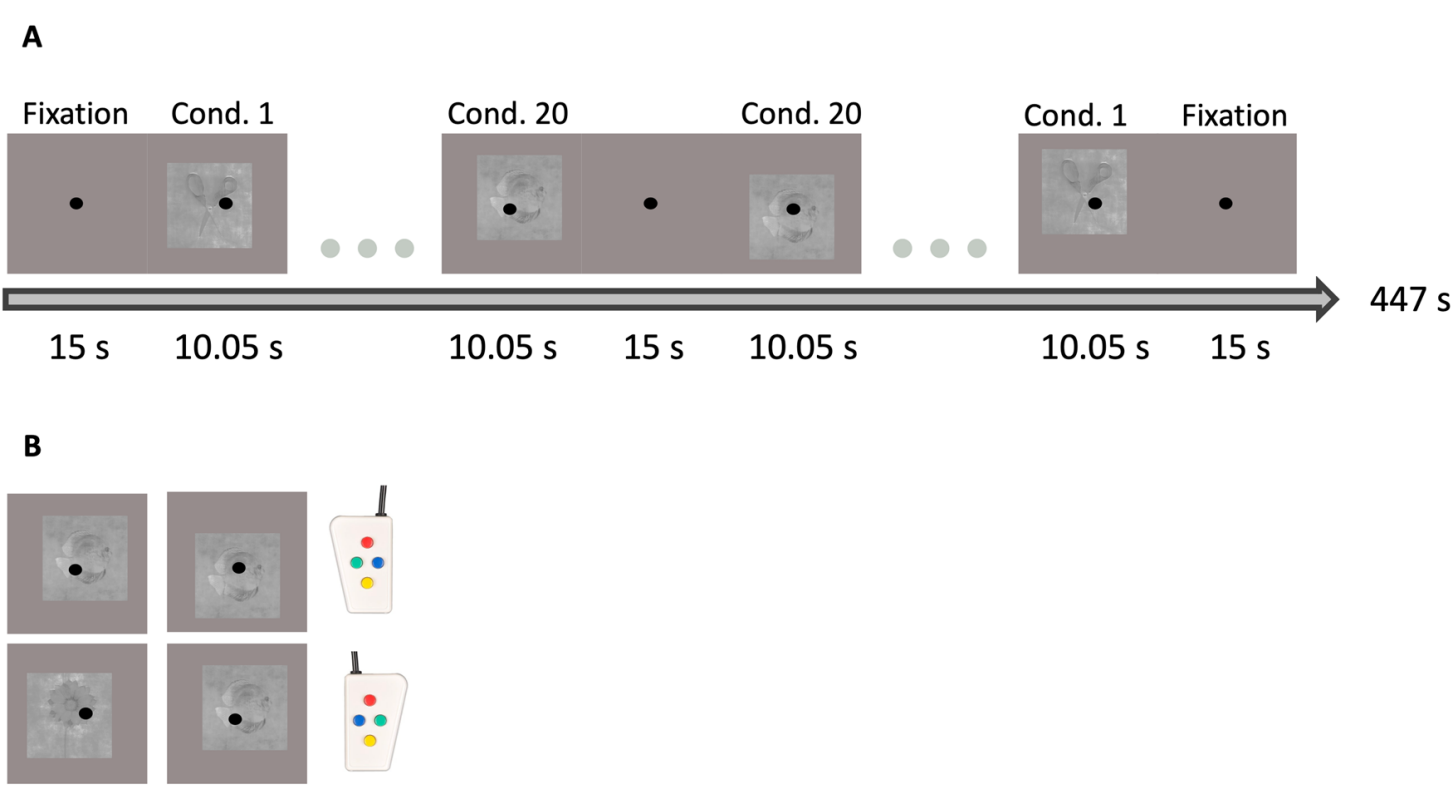
(A) A timeline of the block design of a run of the experiment. (B) Anexample of when the participant had to press the button with theinstructed hand for the one-back task (top row), or the category task(bottom row).

The subjects completed seven runs (one subject only six, sometimes eight) in thescanner. Each run compromised of 40 blocks, each lasting 10.05 seconds andconsisting of 15 trials/stimuli, along with 3 fixation-only blocks (at thestart, middle, and end of the run) that lasted 15 seconds. A run thus lasted 447seconds (seeFig. 2A). After the firstfixation block, the first half of the run presented 20 blocks (one per category)in a random order. After the middle fixation block, the second half of the runpresented the 20 blocks in a reversed order. A trial lasted 0.67 seconds, for40% of the trial (0.27 seconds) only the fixation dot was present, for the other60% of the trial (0.40 seconds) the stimulus was present. Stimuli within a blockwere always presented in a random order, with two stimulus repeats for the firsttask at random points within a block. This resulted in 140.7 seconds of data foreach category per subject with 7 runs. Two types of runs existed: type 1featured images from the first half, while type 2 featured images from thesecond half of the full set of 30 images per category/block.

### 2.4 (f)MRI acquisition

Data were acquired with a 7T Philips Achieva MRI scanner (Philips Healthcare,Best, The Netherlands) with an 8-channel transmit coil and a 32-channel receivecoil (Nova Medical Inc, Wilmington, United States) at the Spinoza Centre forNeuroimaging in Amsterdam (the Netherlands). Functional and anatomical imagingwas carried out using universal pulses (for more explanation, seeGras et al., 2017). During brain imaging,respiratory data were collected using a respiration belt and cardiac data werecollected using a pulse oximeter on a finger of the left hand.

We used a 3D-EPI sequence. The relevant sequence parameters are volume repetitiontime (TR) = 1.37 s, echo time (TE) = 16.9 ms, flip angle =13°, voxel size = 1.79 x 1.79 x 1.8 mm, field-of-view (FOV)= 200 x 200 x 176 mm, and matrix size = 112 x 112 x 98. We alsocollected images with an identical sequence except for reversed phase-encodingblips or in other words, phase-encoding in the opposite direction. Those imagesare used to correct for distortions. For all subjects an anatomical scan wascollected using a MPRAGE sequence with parameters: TR = 10 ms, TE= 3.3 ms, flip angle = 8°, spatial resolution = 0.8x 0.8 x 0.8 mm, and matrix size = 288 x 288 x 205.

### 2.5 Preprocessing

#### 2.5.1 (f)MRI data

The dataset was formatted according to BIDS (Gorgolewski et al., 2016) after converting the PAR/REC files tonifti files using DCM2NIIx (Li et al.,2016) and reorienting them to RAS+ with nibabel in Python.Preprocessing steps were carried out using fMRIPrep 20.2.0 (Esteban et al., 2018;Esteban et al., 2019; RRID:SCR_016216), which isbased on Nipype 1.5.1 (Gorgolewski et al.,2011,^2018^;RRID:SCR_002502). In short, the 7T BOLD images underwent susceptibilitydistortion correction, realignment, coregistration to the T1 weighted image,and spatial normalization. Surface reconstruction with Freesurfer was alsoconducted as part of fMRIprep. Spatially normalized data were not used inour analyses, to preserve the individual subject brain space for maximalspatial and anatomical detail. FMRIPrep’s report providescomprehensive details on the preprocessing steps and can be found below. Inaddition, we looked at tSNR images (average of the time-series divided bythe standard deviation) of every subject to assess the quality of the fMRIdata.

Subjects 4 to 10 experienced a temporary coil issue, causing a small portionof the right hemisphere to appear darker than the left. To mitigate theimpact of this on surface reconstruction of anatomical data, we took severalsteps. For each anatomical scan, the intensity value distribution wasassessed with ITK-SNAP (Yushkevich et al.,2006) and values were clipped off accordingly. The values werethen rescaled using ANTs (Tustison et al.,2014), and denoising and bias correction of the scans was doneusing SPM12 and cat12. Surface reconstruction results (from an fMRIprep anatonly process) were carefully reviewed and improved, ensuring successfulreconstruction of all subjects’ brain surfaces. After this, usingthese results, we ran fMRIprep in full to also conduct functionalpreprocessing steps.

The shadow did not impact functional data preprocessing. Preprocessingresults were thoroughly examined using fMRIPrep reports and by comparingsimilarity of strength and size of contrasts (e.g., faces versus all otherconditions) across different models: (1) a general linear model based on thefunctional space after realignment, (2) a model after realignment,susceptibility distortion correction, and coregistration to T1w space, and(3) a model after all these steps plus normalization to MNI space. Thischeck ensured the effectiveness of all spatial preprocessing steps.

What follows is the detailed description provided by fMRIPrep. A total of 1T1-weighted (T1w) images were found within the input BIDS dataset. TheT1-weighted (T1w) image was corrected for intensity non-uniformity (INU)with N4BiasFieldCorrection (Tustison et al.,2010), distributed with ANTs 2.3.3 (Avants et al., 2008; RRID:SCR_004757), and usedas T1w-reference throughout the workflow. The T1w-reference was thenskull-stripped with a Nipype implementation of the antsBrainExtraction.shworkflow (from ANTs), using OASIS30ANTs as target template. Brain tissuesegmentation of cerebrospinal fluid (CSF), white-matter (WM), andgray-matter (GM) was performed on the brain-extracted T1w using fast (FSL5.0.9, RRID:SCR_002823;Zhang et al.,2001). Brain surfaces were reconstructed using recon-all(FreeSurfer 6.0.1, RRID:SCR_001847;Dale etal., 1999), and the brain mask estimated previously was refinedwith a custom variation of the method to reconcile ANTs-derived andFreeSurfer-derived segmentations of the cortical gray-matter of Mindboggle(RRID:SCR_002438;Klein et al.,2017). Volume-based spatial normalization to one standard space(MNI152NLin2009cAsym) was performed through nonlinear registration withantsRegistration (ANTs 2.3.3), using brain-extracted versions of both T1wreference and the T1w template. The following template was selected forspatial normalization: ICBM 152 Nonlinear Asymmetrical template version2009c [Fonov et al., 2009;RRID:SCR_008796; TemplateFlow ID: MNI152NLin2009cAsym]. For each of thesix/seven/eight (dependent on the subject) BOLD runs found per subject(across all tasks and sessions), the following preprocessing was performed.First, a reference volume and its skull-stripped version were generatedusing a custom methodology of fMRIPrep. A B0-nonuniformity map (or fieldmap)was estimated based on two (or more) echo-planar imaging (EPI) referenceswith opposing phase-encoding directions, with 3dQwarpCox and Hyde (1997)(AFNI 20160207). Based on theestimated susceptibility distortion, a corrected EPI (echo-planar imaging)reference was calculated for a more accurate co-registration with theanatomical reference. The BOLD reference was then co-registered to the T1wreference using bbregister (FreeSurfer) which implements boundary-basedregistration (Greve & Fischl,2009). Co-registration was configured with six degrees offreedom. Head-motion parameters with respect to the BOLD reference(transformation matrices, and six corresponding rotation and translationparameters) are estimated before any spatiotemporal filtering using mcflirt(FSL 5.0.9;Jenkinson et al., 2002).The BOLD time-series were resampled onto the following surfaces (FreeSurferreconstruction nomenclature): fsaverage, fsnative. The BOLD time-series(including slice-timing correction when applied) were resampled onto theiroriginal, native space by applying a single, composite transform to correctfor head-motion and susceptibility distortions. These resampled BOLDtime-series will be referred to as preprocessed BOLD in original space, orjust preprocessed BOLD. The BOLD time-series were resampled into standardspace, generating a preprocessed BOLD run in MNI152NLin2009cAsym space.First, a reference volume and its skull-stripped version were generatedusing a custom methodology of fMRIPrep. Several confounding time-series werecalculated based on the preprocessed BOLD: framewise displacement (FD),DVARS, and three region-wise global signals. FD was computed using twoformulations following Power (absolute sum of relative motions,Power et al., 2014) and Jenkinson(relative root mean square displacement between affines,Jenkinson et al., 2002). FD and DVARS arecalculated for each functional run, both using their implementations inNipype (following the definitions byPoweret al., 2014). The three global signals are extracted within theCSF, the WM, and the whole-brain masks. Additionally, a set of physiologicalregressors were extracted to allow for component-based noise correction(CompCor;Behzadi et al., 2007).Principal components are estimated after high-pass filtering thepreprocessed BOLD time-series (using a discrete cosine filter with 128 scut-off) for the two CompCor variants: temporal (tCompCor) and anatomical(aCompCor). tCompCor components are then calculated from the top 2% variablevoxels within the brain mask. For aCompCor, three probabilistic masks (CSF,WM, and combined CSF + WM) are generated in anatomical space. Theimplementation differs from that ofBehzadiet al. (2007)in that instead of eroding the masks by 2 pixels onBOLD space, the aCompCor masks are subtracted from a mask of pixels thatlikely contain a volume fraction of GM. This mask is obtained by dilating aGM mask extracted from the FreeSurfer’s aseg segmentation, and itensures components are not extracted from voxels containing a minimalfraction of GM. Finally, these masks are resampled into BOLD space andbinarized by thresholding at 0.99 (as in the original implementation).Components are also calculated separately within the WM and CSF masks. Foreach CompCor decomposition, the k components with the largest singularvalues are retained, such that the retained components’ time seriesare sufficient to explain 50 percent of variance across the nuisance mask(CSF, WM, combined, or temporal). The remaining components are dropped fromconsideration. The head-motion estimates calculated in the correction stepwere also placed within the corresponding confounds file. The confoundtime-series derived from head motion estimates and global signals wereexpanded with the inclusion of temporal derivatives and quadratic terms foreach (Satterthwaite et al., 2013).Frames that exceeded a threshold of 0.5 mm FD or 1.5 standardized DVARS wereannotated as motion outliers. All resamplings can be performed with a singleinterpolation step by composing all the pertinent transformations (i.e.,head-motion transform matrices, susceptibility distortion correction whenavailable, and co-registrations to anatomical and output spaces). Gridded(volumetric) resamplings were performed using antsApplyTransforms (ANTs),configured with Lanczos interpolation to minimize the smoothing effects ofother kernels (Lanczos, 1964).Non-gridded (surface) resamplings were performed using mri_vol2surf(FreeSurfer). Many internal operations of fMRIPrep use Nilearn 0.6.2 (Abraham et al., 2014; RRID:SCR_001362),mostly within the functional processing workflow. For more details of thepipeline, see the section corresponding to workflows in fMRIPrep’sdocumentation.

#### 2.5.2 Physiological data

The physiological data were formatted according to BIDS (Gorgolewski et al., 2016) using scanphyslog2bidscreated by Lukas Snoek and available through GitHub (https://github.com/lukassnoek/scanphyslog2bids). Correction forphysiological noise was performed with RETROICOR (Glover et al., 2000;Hutton et al., 2011) using Fourier expansions ofdifferent order for the estimated phases of cardiac pulsation (3rd order),respiration (4th order) and cardio-respiratory interactions (1st order)(Harvey et al., 2008). Thecorresponding confound regressors were created using the MATLAB PhysIOtoolbox (Kasper et al., 2017),open-source code available as part of the TAPAS software collection (https://www.translationalneuromodeling.org/tapas). Thephysiological data collection was not successful for all participants:subjects 2, 3, 4, 5 and the first functional run of subject 18 did notinclude physiological data.

### 2.6 Data analyses

#### 2.6.1 Univariate analysis

The BOLD response of each subject, run, and voxel was modeled using a generallinear model (GLM, also called univariate analysis) with the StatisticalParametric Mapping toolbox (SPM12, Wellcome Centre for Neuroimaging, London)in MATLAB (Mathworks inc.). Within this model, each condition was capturedby a regressor with specified onsets and durations, yielding 21 regressors(20 categories + one fixation condition) per run. Several confoundregressors from either fMRIPrep preprocessing or from the PhysIO toolboxwere also included: the realignment parameters and their temporalderivatives (total: 12) to minimize motion effects on the data andadditional (number differed per run and subject) motion outlier regressors(a binary regressor for each volume labeled as outlier); the resultingconfound regressors from both respiratory and cardiac data (total: 18) whenavailable. A high-pass filter of 610 seconds (based on the design of therun) was applied. Contrasting each category against the average of all othercategories (excluding fixation) generated functional neuroanatomy maps foreach subject. In these maps, we focused on the key categories: faces,bodies, hands, words, numbers, fake script, and a typical objects category(chairs) as a reference landmark. Contrasts were family-wise error corrected(*p*< .05).

All the analyses were conducted within the subject-specific space, thuswithout performing spatial normalization. Following Weiner andGrill-Spector’s advice (2013, summarized in table 2), this approachpreserves the gyral and sulcal patterns of a specific brain for a moreaccurate localization of activity. They also advise against spatialsmoothing to prevent inaccurate activity localization and averaging togetherregions that are in truth distant on the surface. We adhere to the pipelineoutlined byBrodoehl et al. (2020),conducting GLM analysis without spatial normalization or smoothing in thevolume-space, with results projected onto the brain surface.

##### 2.6.1.1 Split-half analysis

To quantify and demonstrate the replicability of category selectivity, weconducted a split-half analysis. First, we defined a region of interest(ROI) on the left and right hemisphere surface of each subject,encompassing the middle-anterior ventral surface of the OTC (activityanterior to the inferior occipital gyrus, including the fusiform gyrusand neighboring occipitotemporal sulcus, seeFig. 3A). This ROI was designed to include allsecond and third clusters of selectivity for words, faces, and hands,bodies, numbers, fake script, and objects (chairs) (see Results). Onesubject (10) lacked these activity clusters in the right hemisphere, sono ROI was drawn here. We chose only the middle and anterior ventralsurface to create these ROIs, to investigate the response profile ofword areas (compared to other category-selective areas) that show moreselectivity to words than to other characters (this was also confirmedlater by the results from this analysis). Based on previous research,posterior activity is less selective to words and more selective tocharacters in general (this is also visible on the surface maps from theresults section). Therefore, posterior activity was not included for anyof the ROIs.

**Fig. 3.**
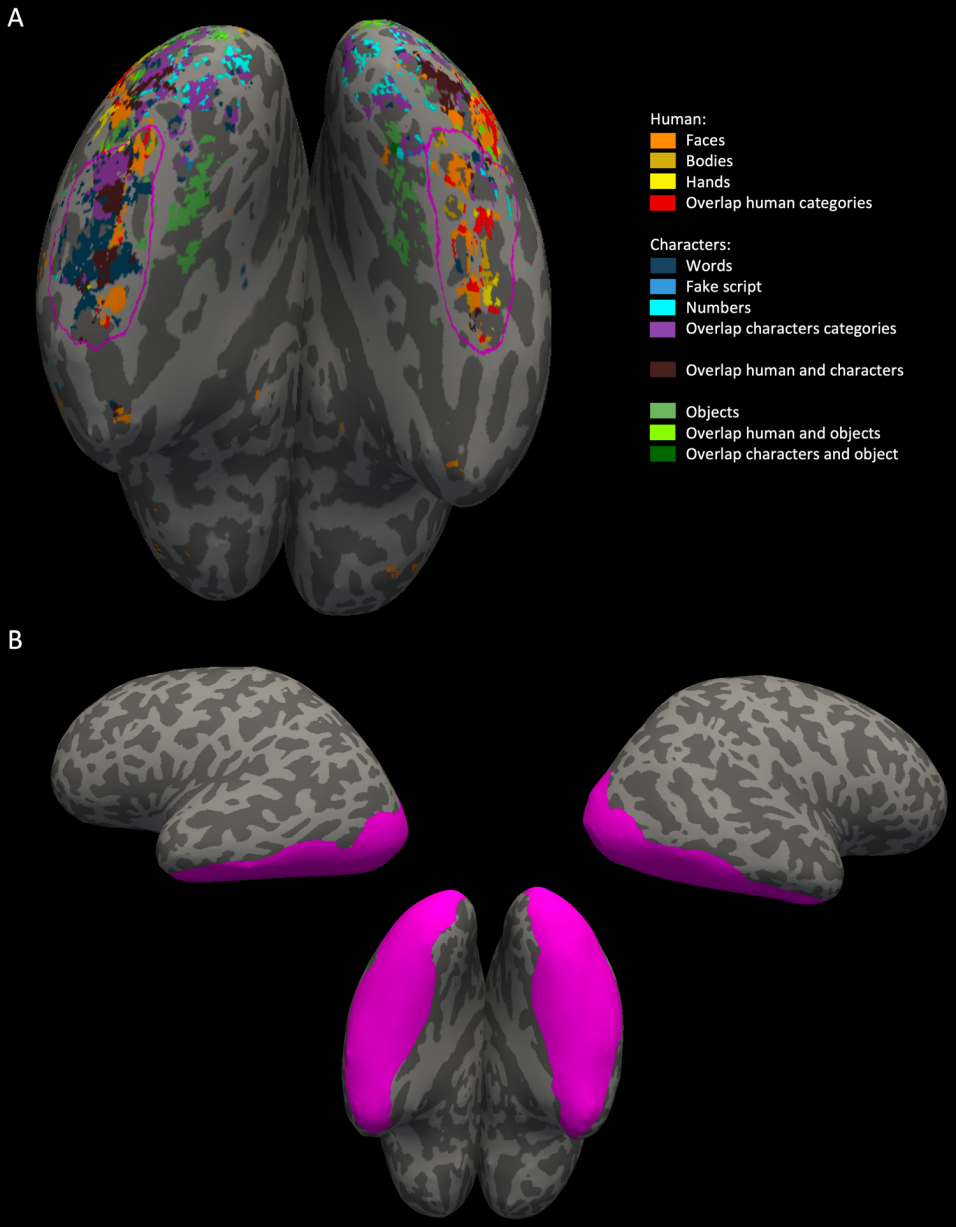
(A) The region of interest (ROI) for the split-half analysis forboth the left and right hemisphere, shown using a pink outlineon a brain surface of one of the participants. The ROI includesall middle and anterior selectivity to words (dark blue on thesurface), faces (orange) and hands (yellow). (B) The region ofinterest (ROI) for multi-voxel pattern analysis, here shown forboth the left and right hemisphere of the occipitotemporalcortex (OTC) in pink on a brain surface of one of theparticipants. The left and right OTC ROI was created separatelyby combining the lateral occipital cortex, inferior temporalgyrus, and fusiform gyrus from the Desikan-Killiany atlasincorporated in FreeSurfer.

Second, for every subject, we ran two GLMs: one with the odd runs and onewith the even runs. Using the odd runs GLM, we computed the contrasts ofinterest, including one category (faces, bodies, hands, words, numbers,fake script, or an objects category (chairs)) versus all other 19categories. These contrasts were set with an uncorrected*p*< .0005. Per subject, we intersected eachfunctional contrast with the left and the right hemisphere anatomicalROI. As a result, 7 anatomical-functional ROIs were created perhemisphere for each subject: a ventral middle-anterior face, body, hand,word, number, fake script, and object area. It was not possible tocreate every ROI for every subject (due to no significant voxels duringintersecting).

Third, using the even runs GLM, we selected the beta values of all 21conditions per ROI and per subject, and averaged them across all runsand all voxels within an ROI. This yielded one beta value per category,per ROI, and per subject. Finally, we averaged across subjects to createa bar graph depicting the averaged beta value per category per ROI andcalculated the standard error for each category/bar.

Within each ROI, we selected the main relevant category for thefunctional contrast defining that ROI (e.g., faces for the left ventralmiddle-anterior face area). Using a paired t-test, we compared thiscategory to the other categories of interest resulting in 6 tests perROI (e.g., for the face area this would be faces against either bodies,hands, words, fake script, numbers, and chairs). These tests wereBonferroni corrected per ROI (*p*< .008). We alsoperformed a paired t-test on the difference between the first and secondpreferred category between the hand- and body-selective voxels in theleft hemisphere. We also performed several paired t-tests on thedifference of two categories between the left and right hemisphere ROIs:between the word areas for the difference between words and numbers andfor the difference between words and fake script (Bonferroni-corrected*p*< .03) and between the hand areas for thedifference between hands and words.

#### 2.6.2 Multi-voxel pattern analysis (MVPA)

FreeSurfer performs an automated aparc parcellation using theDesikan-Killiany atlas. From this parcellation, we constructed a large OTCROI separately for each hemisphere, by including the fusiform gyrus,inferior temporal, and lateral occipital cortical regions according to theaforementioned parcellation (e.g.,Lerma-Usabiaga et al., 2018;Mattioni et al., 2020; seeFig.3B). We used the CoSMoMVPA toolbox (Oosterhof et al., 2016) for MATLAB (Mathworks,Inc.). A multi-voxel pattern in response to each of the conditions for eachrun was constructed by using the beta coefficient estimates for all voxelspresent within the ROI. We used the cross-validated Mahalanobis distance(Walther et al., 2016), alsocalled linear discriminant contrast (LDC), to decode the condition for everypossible pair of conditions (excluding the fixation condition), such asfaces versus scenes, words versus numbers. The code for this was written byJ. Brendan Ritchie. The results were presented in a so-called dissimilaritymatrix where each point in the matrix reflected the distance between thecondition linked to that row and the condition linked to that column of thematrix. The distance signified a measure of dissimilarity between themulti-voxel pattern of the ROI in response to the row-condition versus thecolumn-condition. The higher the distance, the more dissimilar the patternsof these two conditions were. The distance estimates for each condition pairwere created using a cross-validation scheme where the data are split upinto training and testing folds according to the standard leave-one-run-outpartitioner. Each run was used as the testing fold while all others wereused for the training fold once, generating as many distance estimates asthere were runs available for that subject. These estimates were thenaveraged, and this average was placed in the appropriate spot in thedissimilarity matrix. Then, we normalized each subject matrix, per ROI, bydividing all the values inside by the maximum value of that matrix.Consequently, we averaged the matrix across subjects per ROI. To show thereliability of these matrices, per ROI, each subject matrix was correlatedwith the average (calculated without that subject) matrix. We performedseveral paired t-tests (with a Bonferroni-corrected*p*-value) between different distances, to confirmobservations made from the multidimensional scaling plots (describedbelow).

##### 2.6.2.1 Multidimensional scaling (MDS) and Procrustes transformations

Multidimensional scaling (MDS) was used to visualize the main dimensionsunderlying the patterns in the representational dissimilarity matricesin a two-dimensional space where the distance between the points in thisspace was a measure for how dissimilar these points were: the higher thedistance, the more dissimilar. We used the built-in MATLAB (Mathworks,Inc.) function mdscale with the default parameters, while minimizing thedefault goodness-of-fit criterion: stress, and using a 100 replicates ofthe scaling. We applied MDS on the matrices, averaged across subjects,keeping left and right OTC separate. In addition, we also applied MDS onthe normalized dissimilarity matrices of each individual participant.The MDS results of every subject, per ROI, were transformed to theaverage MDS results using a Procrustes transformation (using thebuilt-in MATLAB (Mathworks, Inc.) function procrustes). We thenvisualized the average MDS results in a 2D space per ROI. In this spaceper ROI, for each category, 19 lines were drawn using theProcrustes-transformed individual subject MDS position of that category.The line started in the dot of a category and ended at the coordinates(Procrustes-transformed MDS results) of that category for the individualparticipant.

## 3 Results

### 3.1 Functional neuroanatomy of ventral OTC

#### 3.1.1 Widespread and abundant activity to all categories of interest

**Fig. 4.**
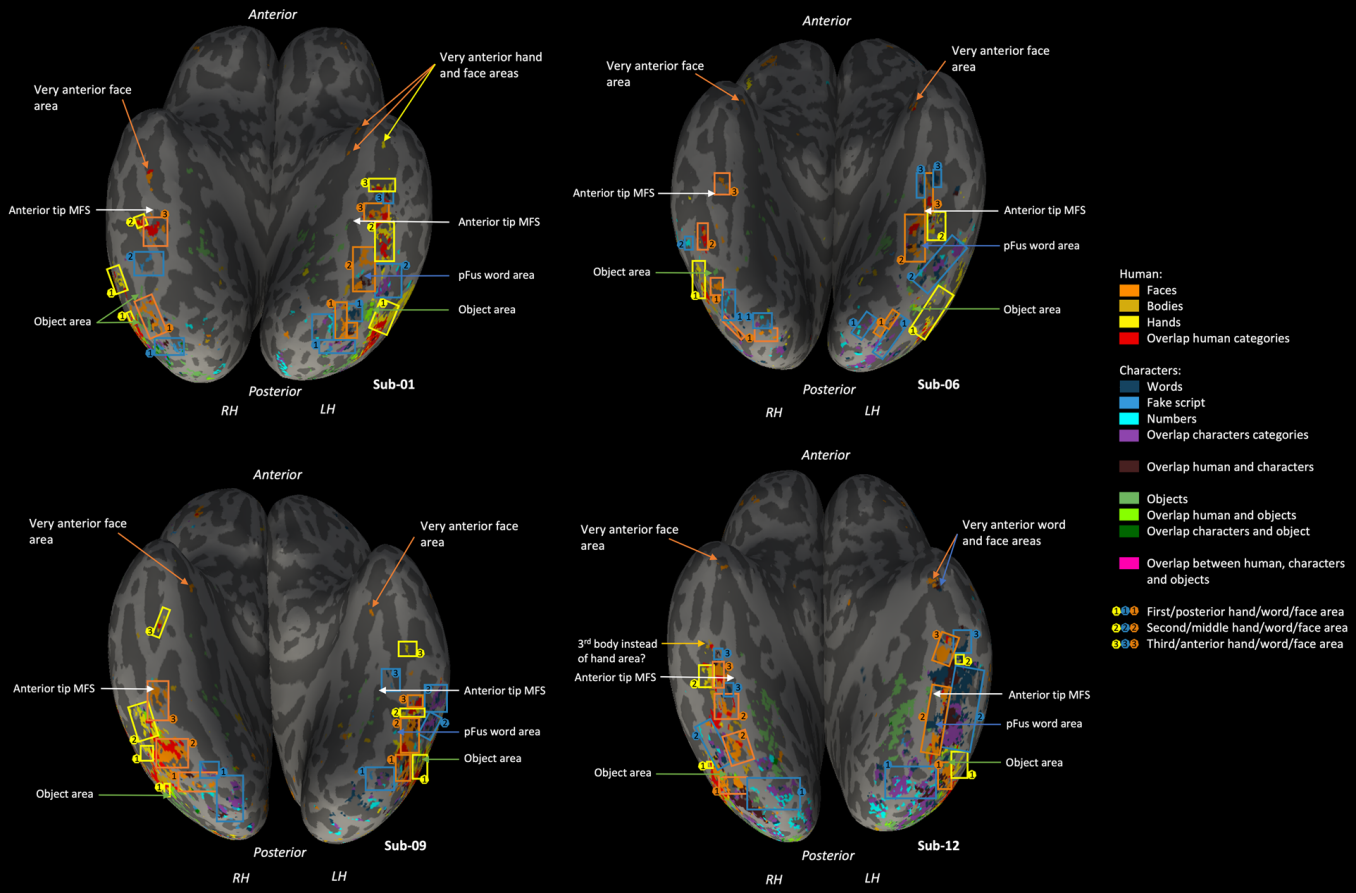
Category-selective regions shown upon annotated right (RH) and left(LH) hemisphere ventral surfaces of four example participants,together with an annotation of how to structure this categoryselectivity in three clusters of hand, word, and face selectivity.Annotations were made using a circle, each linked to a square, withthe number 1, 2, or 3 to indicate the first, second, or third hand(yellow), word (dark blue), and face (orange) area. They alsoinclude several arrows that point to the posterior object region (ingreen), the pFus word area (dark blue), the anterior tip of themid-fusiform sulcus (white), a possible third body instead of thethird hand area (ochre), and lastly, arrows pointing to even moreanterior areas (color depends on the category). Color legend on theright. Selective activation (contrast of one versus all othercategories except fixation,*p*< .05, FWEcorrected) to faces in orange, to bodies in ochre, to hands inyellow, to words in dark blue, to fake script in medium blue, tonumbers in light blue, and to an objects category (chairs) in mediumgreen. Overlap between selectivity for different human-relatedcategories in red, overlap between selectivity for differentcharacter categories in purple, overlap between selectivity forhuman-related categories overlapping with character categories inbrown, overlap between selectivity for human-related categoriesoverlapping with selectivity for the objects category in lightgreen, overlap between selectivity for character categoriesoverlapping with the objects category in dark green, and overlapbetween selectivity for human-related characters and the objectscategory in pink.

We created a map of the functional neuroanatomy in response to the mainconditions in our experiment. Each subject’s brain surface wasreconstructed and results from various contrasts, calculated using the GLM,were projected onto this surface.Figure4shows four exemplary subject surfaces (surfaces of all subjectsare available inSupplementary Fig. 2). On each surface, selective activation(contrast of one versus all other categories except fixation,*p*< .05, FWE corrected) to several categorieswas displayed: to faces (orange), to bodies (ochre), to hands (yellow), towords (dark blue), to fake script (medium blue), to numbers (light blue),and to a typical objects category (chairs, medium green) to provide areference landmark.

Before focusing on words, we noted some general findings. First, concerningcategory selectivity, it was present for each main category in all oursubjects, thus for all types of characters and for all types ofhuman-related categories (seeFig. 4).This category selectivity was extensive and widespread. The human-relatedand character category selectivity appeared on the lateral ventral surface,whereas object selectivity appeared more medial, except in the moreposterior parts (posterior to the fusiform gyrus, within the inferioroccipital gyrus) of the ventral surface (seeFig. 4). This category selectivity was grouped into separateareas (these areas are described in the following sections). The way theseareas were grouped was based on a consistent pattern of selectivity foundacross participants. This pattern is described in detail inSection 3.1.2.1and is also included inthe Supplementary Results. Sometimes, participants showed some deviationfrom this standard pattern. In these cases, we used this standard pattern asmuch as possible to make decisions about which activity to include intoareas, but nonetheless these decisions are somewhat uncertain and subjective(examples are given in theSupplementary Results). This variability was the mostobvious when certain areas were missing, and this type of variability wasmade explicit in the following results sections by mentioning which subjectsdigressed from the standard (and this is also summarized inSupplementary Fig.1).

Second, we noted several findings concerning overlaps between categoryselectivity. InFigure 4, overlapbetween selectivity for different human-related categories is depicted inred, for different character categories in purple, and for human-relatedcategories overlapping with character categories in brown. These types ofoverlaps appeared all over the ventral surface in a predictable manner (seeFig. 4): where selectivity fordifferent human-related categories was nearby, often clusters of overlapbetween different human-related category selectivity emerged (shown in red).These seemed to act as a transition between the different categoryselectivity. This was similar for overlap between selectivity for charactercategories (shown in purple). This was also similar for the overlap betweenselectivity for human-related and character categories (shown in brown):when selectivity for human-related and character categories appeared neareach other, we often also found this type of overlap, suggesting atransition between the two kinds of selectivity. Overlap between selectivityfor human-related categories overlapping with selectivity for the objectscategory is depicted in light green, and for character categoriesoverlapping with the objects category in dark green. These kinds of overlapappeared in a small amount in the posterior parts (posterior to the fusiformgyrus, within the inferior occipital gyrus) of the ventral surface (see,e.g., subject 14 inSupplementary Fig. 2). The overlap between all types ofselectivity (to human-related characters and the objects categories) isdepicted in pink. As expected, given that each category selectivity wasdefined as one versus all other categories, this type of overlap was rarelypresent and if so, it was very small (see, e.g., subject 1 inFig. 4).

#### 3.1.2 Several word-selective areas exist among areas selective to faces, hands, and bodies in the left hemisphere

To discern the spatial organization of word selectivity, we firstinvestigated where word selectivity (also called the visual word form area(VWFA)) was located on the left hemisphere ventral surface and if we couldconsistently divide this activity into separate regions. Then, weinvestigated if this organization was consistent across subjects. Second, welocated an important landmark in each subject (Weiner et al., 2014): the left hemispheremid-fusiform sulcus (MFS). The anterior tip of this sulcus can predict thelocation of the FFA-2 or mFus face area, located on the middle fusiformgyrus (Weiner et al., 2014). Bylocating the MFS, we could investigate if this landmark also provedimportant for locating word selectivity. Third, we explored if theorganization of word selectivity might be consistently related to thelocation of other category selectivity. We refer toFigure 4for all anatomical descriptions writtenbelow.

First, we consistently observed three clusters of word selectivity on theleft hemisphere ventral surface of almost every subject (subject 4, 7, and17 lacked the most anterior cluster and 17 also lacked the posteriorcluster,SupplementaryFig. 1gives an overview of which areas were found in whichsubjects). The first cluster was located posterior to the fusiform gyrus,within the inferior occipital gyrus, the second one more anterior within theposterior part of the fusiform gyrus and the occipitotemporal sulcus, andthe third even more anterior, within the anterior part of the fusiform gyrusand sometimes including the anterior part of the occipitotemporal sulcus.The second cluster appeared in between the first/posterior andthird/anterior cluster but was often located more lateral than them,towards/on the occipitotemporal sulcus. To quantify the replicability andsize of the word selectivity, we performed a split-half analysis focusedupon the middle and anterior ventral surface. The region of interest thuscovered the second and third cluster of selectivity to words, faces, andhands. We were able to identify (in 15 subjects) word-selective voxels usinghalf of the scans. Across these voxels, activity (as quantified by the betavalue, see Methods section) to words in the other half of the scans wassignificantly (i.e.,*p*< .008 based on Bonferronicorrection) higher than to any of the other main categories of interest (seeFig. 5A, for statistical detailsseeSupplementary Table1). The word-selective voxels showed a significantly higherresponse to words than to numbers and to fake script, even though the sizeof these differences was clearly smaller than when words were compared withobjects categories. The division of this word selectivity into threesubregions was helped by relating it to the selectivity for othercategories. These categories are discussed in the following sections.

**Fig. 5.**
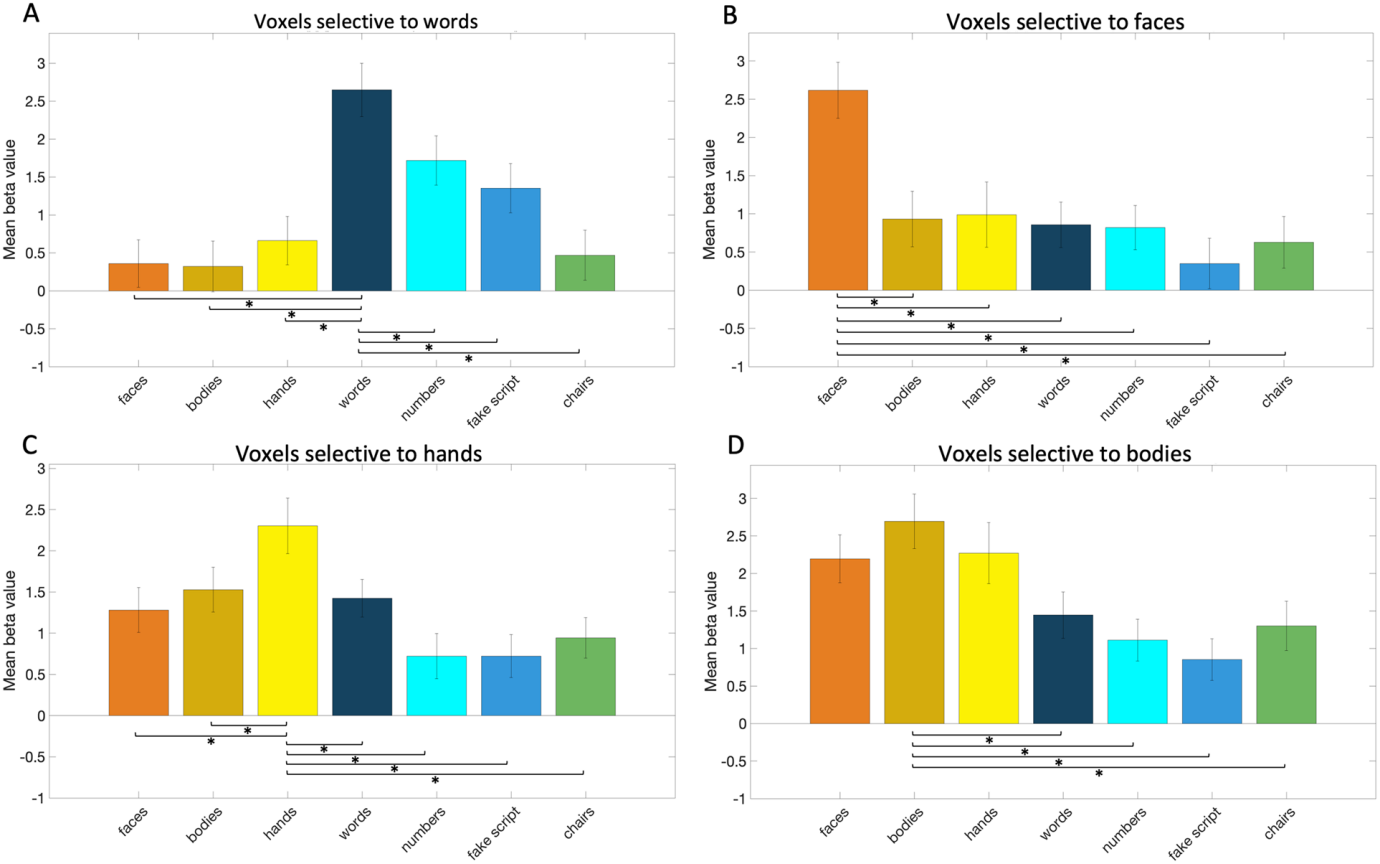
The response in word- (A), face- (B), hand- (C), and body- (D)selective voxels of the left hemisphere to seven categories (faces,bodies, hands, words, numbers, fake script, and objects: chairs).Responses were calculated using data that were independent from thedata used to select the voxels (see Methods). Error bars representthe standard error, and the lines and stars indicate which pairedt-tests between conditions were significant (*p*< .008).

Second, we assessed the relevance of the mid-fusiform sulcus (MFS) to locatethe different word-selective clusters on top of its relevance for faceareas. In most subjects, the selectivity for faces was plentiful anddistributed over most of the ventral surface. First, we determined thelocation of the MFS (Weiner et al.,2014). Second, we identified the three face areas in the lefthemisphere, as described inWeiner andGrill-Spector (2010): in the inferior occipital gyrus (IOG); FFA1or pFus, located in the posterior fusiform gyrus; and FFA2 or mFus, locatedin the mid-fusiform gyrus. This was possible in almost all subjects (subject13’s anterior cluster seemed too anterior to be mFus, subject 14lacked the pFus, and subject 15 and subject 17 lacked the IOG). In somesubjects, the separation between pFus and mFus was not straightforward(subject 16 and subject 19). The replicability of face selectivity overallwas tested with the split-half analysis. We were able to identify (in 14subjects) face-selective voxels across the middle and anterior ventralsurface with one half of the scans. Across these voxels, activity (asquantified by the beta value, see Methods section) to faces in the otherhalf of the scans was significantly (i.e.,*p*< .008based on Bonferroni correction) higher than to any of the other maincategories of interest (seeFig. 5B,for statistical details seeSupplementary Table 1). Third, we investigated therole of the MFS in locating the subregions of the VWFA. Its contribution wasnot critical but a few relations were often present. We noticed that thesecond/middle subregion of the VWFA was lateral and more posterior relativeto the anterior tip of the MFS. Additionally, the anterior subregion of theVWFA appeared in the neighborhood of the mFus, and this mFus face area wasoften in line with the anterior tip of the MFS as previously demonstrated byWeiner and Grill-Spector (2010).However, the anterior word area was often located further away (relative tothe mFus face area) from the anterior tip of the MFS.

Third and finally, we looked at the selectivity to the other categoriesbesides words and faces. We found that there were at least two, sometimesthree (in subject 1, 9, 10, 13, and 19), clusters of selectivity to hands.The first one appeared within the inferior occipital gyrus and lateraloccipital sulcus. The second one was located along the middle of thefusiform gyrus, sometimes including the neighboring occipital temporalsulcus. The third one, when present, appeared anterior in the fusiformgyrus. On the surfaces of some subjects (subject 3, 7, 8, and 18), we couldnot determine the third anterior hand cluster, but instead they did show ananterior cluster of selectivity to bodies where we expected the third handcluster. The replicability of hand selectivity overall was tested with thesplit-half analysis. We were able to identify (in 16 subjects)hand-selective voxels across the middle and anterior ventral surface. Acrossthese voxels, activity (as quantified by the beta value, see Methodssection) to hands was significantly (i.e.,*p*< .008based on Bonferroni correction) higher than to any of the other maincategories of interest (seeFig. 5C,for statistical details seeSupplementary Table 1). The hand-selective voxelsshowed a significantly higher response to hands than to faces and to bodies,but the size of these differences was clearly smaller than when hands werecompared with character categories (with the exception of the wordscategory) or objects categories.

Considering that body selectivity has often been used to obtain a detailedpicture of face selectivity (e.g.,Weiner& Grill-Spector, 2010), we looked at the body-selectiveareas and found them to lie separate from the hand areas. We also identifiedbody-selective voxels across the middle and anterior ventral surface usingthe split-half analysis. Across these body-selective voxels (identified in15 subjects), activity (as quantified by the beta value, see Methodssection) to bodies was significantly (i.e.,*p*< .008based on Bonferroni correction) higher than to words, numbers, fake script,and objects (chairs) (seeFig. 5D, forstatistical details seeSupplementary Table 2). However, activity to bodies was notsignificantly different from faces (significant only at uncorrectedthreshold:*p*< .05) or hands. In contrast, in thehand-selective voxels, the activity to hands was significantly higher thanto faces or bodies (described above). To compare the activity to itspreferred category and second-preferred category, we compared the hand- andbody-selective voxels/areas in a paired t-test. Specifically, we comparedthe difference between bodies and hands in the body area with the differencebetween hands and bodies in the hand area. We did not find a significantdifference, although there was a trend (*t*(12) =-1.45,*p*= .17): the difference between bodies andhands in the body area tended to be smaller than the difference betweenhands and bodies in the hand area. This is all the more reason to considerhand selectivity as an important property in ventral occipitotemporalcortex.

With the split-half analysis, we could also identify voxels selective tonumbers and to fake script across the middle and anterior ventral surface,but there was no evidence for more selectivity to numbers than words, or tofake script than words, in the number and fake script areas. First, acrossthese number-selective voxels (identified in 17 subjects), activity (asquantified by the beta value, see Methods section) to numbers wassignificantly (i.e.,*p*< .008 based on Bonferronicorrection) higher than to faces, bodies, hands, fake script, and objects(chairs) (seeSupplementary Fig. 3A, for statistical details seeSupplementary Table2). It was not significantly different from words. Second, acrossthese fake script-selective voxels (identified in 17 subjects), activity (asquantified by the beta value, see Methods section) to fake script wassignificantly (i.e.,*p*< .008 based on Bonferronicorrection) higher than to faces, bodies, hands, and objects (chairs) (seeSupplementary Fig.3B, for statistical details seeSupplementary Table2). It was significantly lower than words and not different fromnumbers. Lastly, given that on the map of the ventral surface, we usedselectivity to a typical objects category (chairs) as a reference landmark,we also attempted to identify object-selective voxels using the split-halfanalysis. We identified these object-selective voxels in all 19 subjects andfound that activity (as quantified by the beta value, see Methods section)to objects was significantly (i.e.,*p*< .008 basedon Bonferroni correction) higher than to words, numbers, and fake script,but not different (significant only at uncorrected threshold:*p*< .05) from faces, bodies, and hands (seeSupplementary Fig.3C, for statistical details seeSupplementary Table2).

##### 3.1.2.1 The location of hand selectivity serves as a reference point to locate word and face selectivity in the left hemisphere

The clusters of selectivity to hands were consistent in location andserved as an important reference point to locate the subregions of theVWFA and the typical face areas (IOG, pFus, and mFus) in most subjects.First, for the posterior part of the ventral surface within the inferioroccipital gyrus and sometimes including lateral occipital sulcus, wefound that the first/posterior hand region was important. Thishand-selective area was previously described byBracci et al. (2010)as a region separatefrom the extrastriate body area (EBA) and selective to hands more thanto whole bodies and other body parts. Adjacent to this first handregion, more medial on the inferior occipital gyrus, we found aface-selective cluster (IOG) and a first word-selective cluster ofactivity, around this IOG face area. In many of the subjects, on top ofthis word cluster around IOG, there was even more activity to words andother character categories distributed across the posterior ventralsurface (including the inferior occipital gyrus), but still on the moremedial side of this first hand region. In some subjects, we also foundthat a cluster of selectivity to objects and/or a cluster of overlap ofobject selectivity with character and/or human-related selectivity,separated this first hand cluster from the first face and word cluster(the most clearly visible in subject 1, 3, 6, 9, 11, 12, and 18).

**Fig. 6.**
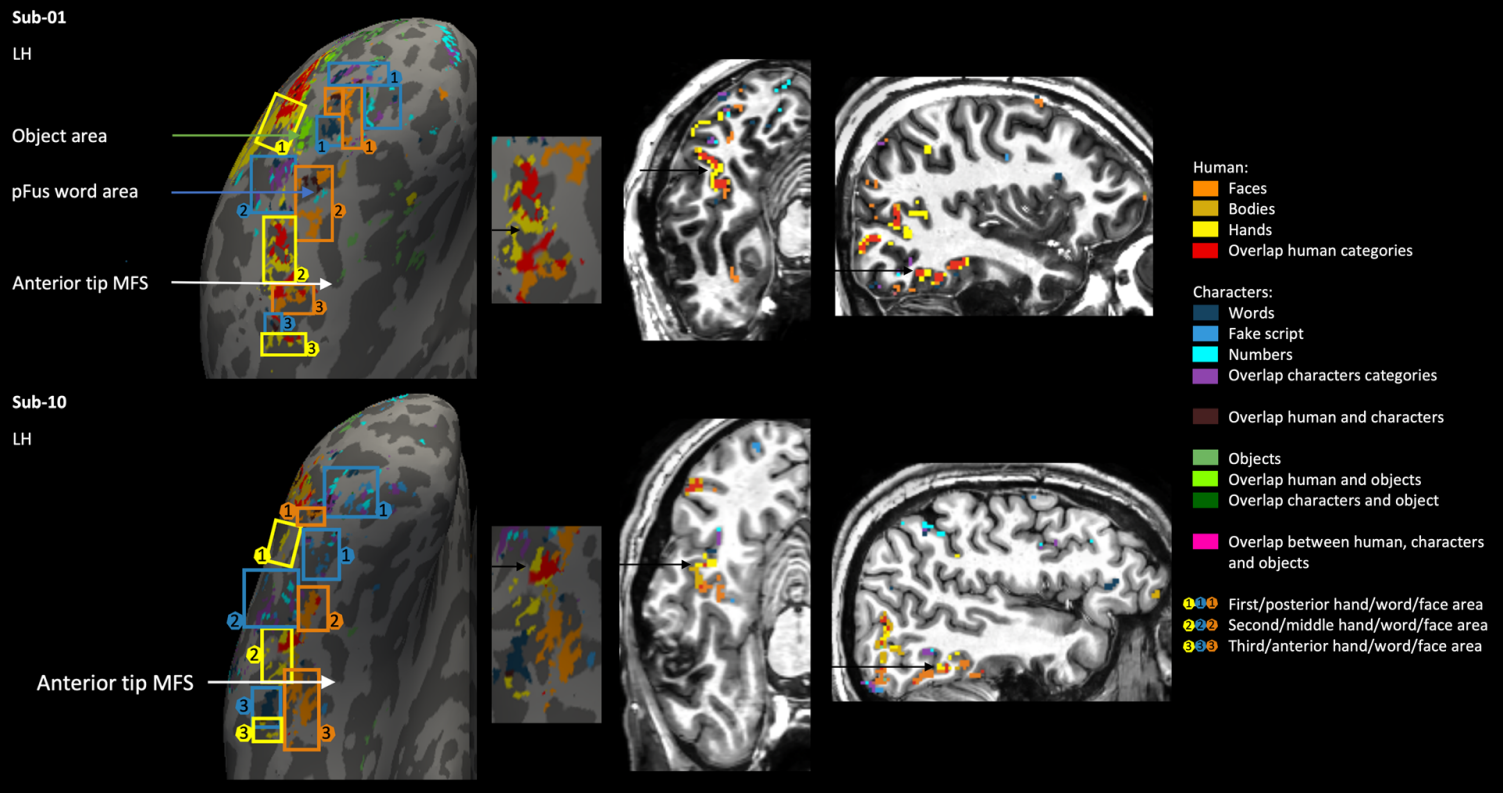
Visualization of the second/middle hand area of subject 1 (top)and subject 10 (bottom) in surface space on the left, next to ita zoomed-in visual of the middle ventral surface, and then onthe right the corresponding volume space in axial and sagittalview. Black arrows on the zoomed-in surface in the middleindicate the location used to visualize the volume space on theright (this location is also indicated by black arrows on thevolume on the right).

**Fig. 7.**
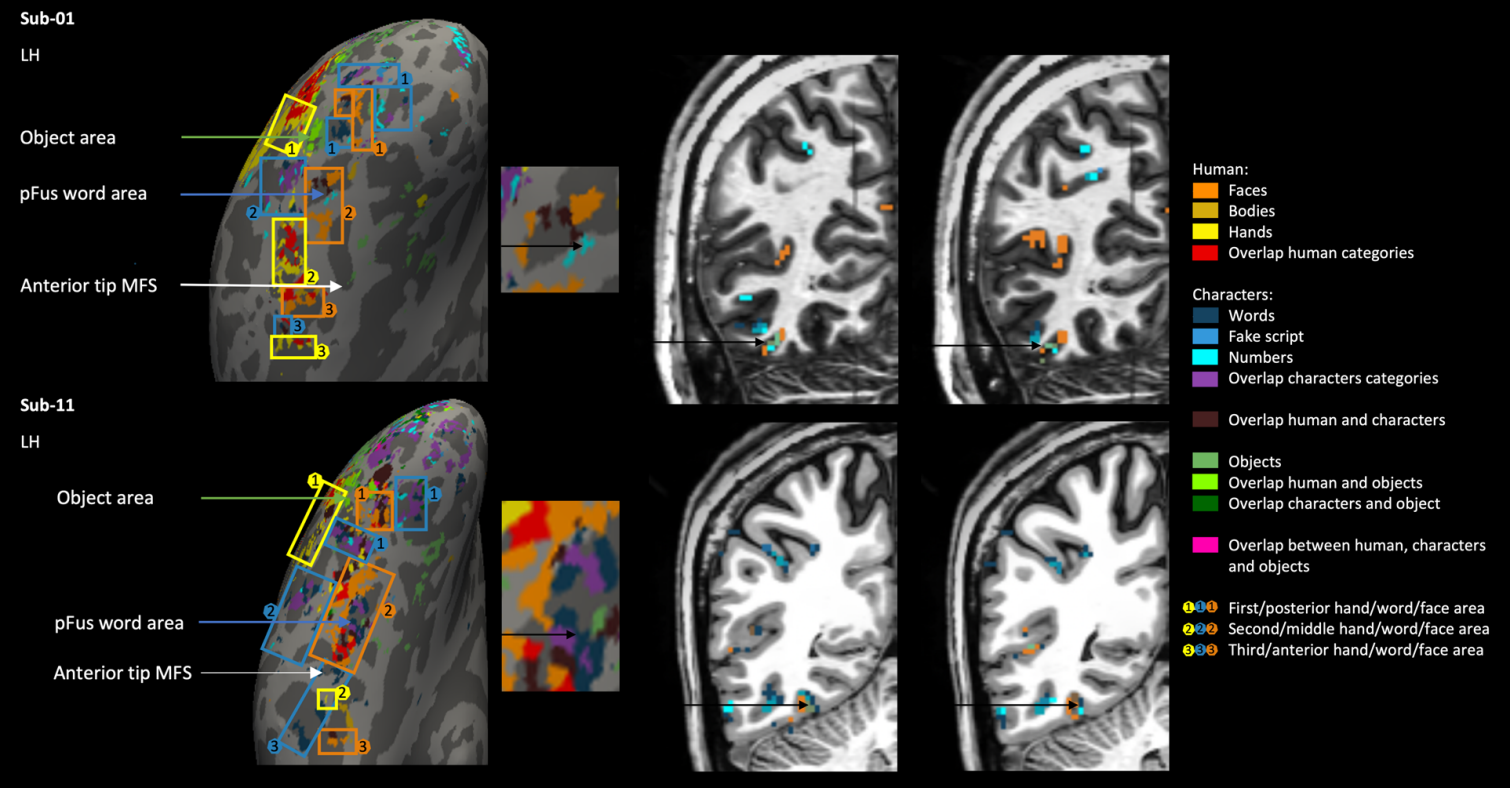
Visualization of the pFus word area of subject 1 (top) andsubject 11 (bottom) in surface space on the left, next to it azoomed-in visual of the middle ventral surface, and then on theright the corresponding volume space in two adjoining slices incoronal view. Black arrows on the zoomed-in surface in themiddle indicate the location used to visualize the volume spaceon the right (this location is also indicated by black arrows onthe volume on the right).

Second, for the middle part of the ventral surface along the fusiformgyrus and sometimes including the occipitotemporal sulcus, thesecond/middle cluster of selectivity to hands also proved to be a usefulreference landmark. In between the first and second cluster of handselectivity, the second word-selective cluster of activity was located(along the middle occipitotemporal sulcus and fusiform gyrus), with moremedial to that word cluster, the second face selectivity cluster (pFus).Due to the central location of the second selectivity cluster to hands,we decided to examine this selectivity also in the volume space (Fig. 6). We found that allselectivity to categories on and around this location of the second handcluster lined up with the anatomical structure of the brain. We alsofound that pFus/FFA1 seemed to be joined, or sometimes broken up intotwo, by another cluster of selectivity to words and/or other characters(visible in subject 1, 2, 3, 4, 5, 6, 7, 8, 9, 11, 12, 15, and 16). Wecalled this the pFus word area. To investigate this more closely, wealso looked at this area in the volume space (Fig. 7) in two of our subjects. In subject 1, thepFus word area seemed to break the pFus area in two, whereas in subject11, the pFus word area adjoined the pFus area.

Third, we looked at the anterior part of the fusiform gyrus andoccipitotemporal sulcus on the ventral surface. Like mentioned above, ina couple of the subjects, there seemed to be a small third/anteriorcluster of selectivity to hands and/or bodies. Around the second handcluster, in between the second and the third hand selectivity cluster(when present), we found the third face (mFus) and word selectivitycluster. Looking even more anterior than these areas, we also regularlyfound very anterior face selectivity clusters: in subject 1 (accompaniedby a hand selectivity cluster), 3, 4 (+ hand cluster), 5(accompanied by a word selectivity cluster), 6, 9, 10, 12 (+ wordcluster), 13 (if this is not in fact mFus, see above, + wordcluster), 16, and 19.

#### 3.1.3 Less word selectivity in the right hemisphere

Previous research observed the most prominent selectivity to words in theleft hemisphere and thus studies have often analyzed only the lefthemisphere. After investigating the left hemisphere of our subjects, wesought out to compare it to the selectivity found in the right hemisphere.Specifically, we compared if the standard organization of the lefthemisphere selectivity clusters was similar in the right hemisphere. Thisconsisted of first checking if all the same types of selectivity werepresent and if the same number of clusters of each type of selectivityappeared. Second, we examined if these areas were organized in a similarway. We refer toFigure 4for allanatomical descriptions written below. Overall, we found the same types ofselectivity in the right hemisphere as in the left hemisphere. Some types ofselectivity were often less clear in appearance in the right versus the lefthemisphere (e.g., smaller, more distributed, or a somewhat strange orinconsistent location in many subjects).

We were able to consistently find the posterior and middle word areas (exceptin subject 9, 10, 11, 16, 18, and 19) of the ventral surface. In severalsubjects however, this selectivity did not appear selectivity to wordsspecifically but rather to characters in general (posterior area: seesubject 1, 5, 8, 14, 16, and 18; middle area: see subject 1, 2, 6, 8, and14). This was also evidenced by the split-half analysis, described below,that showed no difference between selectivity to words, numbers, or fakescript within the word-selective voxels of the middle and anterior ventralsurface. The third word area could only be found in some subjects (subject5, 12, and 18). In subject 2, 8, and 9, there was instead a smallselectivity cluster to the numbers character category and in subject 2,there was also a cluster of selectivity to numbers, but more lateral thanexpected based on the left hemisphere organization. The replicability ofword selectivity overall was tested with the split-half analysis, and wewere able to identify (in 11 subjects) word-selective voxels across themiddle and anterior ventral surface (seeFig.8A, for statistical details seeSupplementary Table1). Across these voxels, activity (as quantified by the betavalue, see Methods section) to words was significantly (i.e.,*p*< .008 based on Bonferroni correction) higherthan to faces, and bodies, but not different from (significant only atuncorrected threshold*p*< .05) hands, fake script,and objects/chairs. It was also not significantly different from numbers. Tofurther look into the lack of difference in selectivity between words,numbers, and fake script in these right-hemisphere word-selective voxels, asopposed to the left-hemisphere word-selective voxels, we performed twopaired t-tests to directly compare the two hemispheres. We appliedBonferroni correction at*p*< .03. In the first test,we compared the difference between words and numbers between the left andright hemisphere word-selective voxels; in the second, we compared thedifference between words and fake script. Of note to these comparisons isthat in the left hemisphere, we could not identify word-selective voxels in4 of the 19 subjects, whereas in the right hemisphere we could not identifysuch voxels in 8 of the 19 subjects. We found that in the left hemisphere,the difference between words and numbers (*t*(7) =2.6,*p*= .04), and between words and fake script(*t*(7) = 3.78,*p*= .007),was significantly bigger (marginally unsignificant in case of words versusnumbers) as compared to the right hemisphere word-selective voxels.

**Fig. 8.**
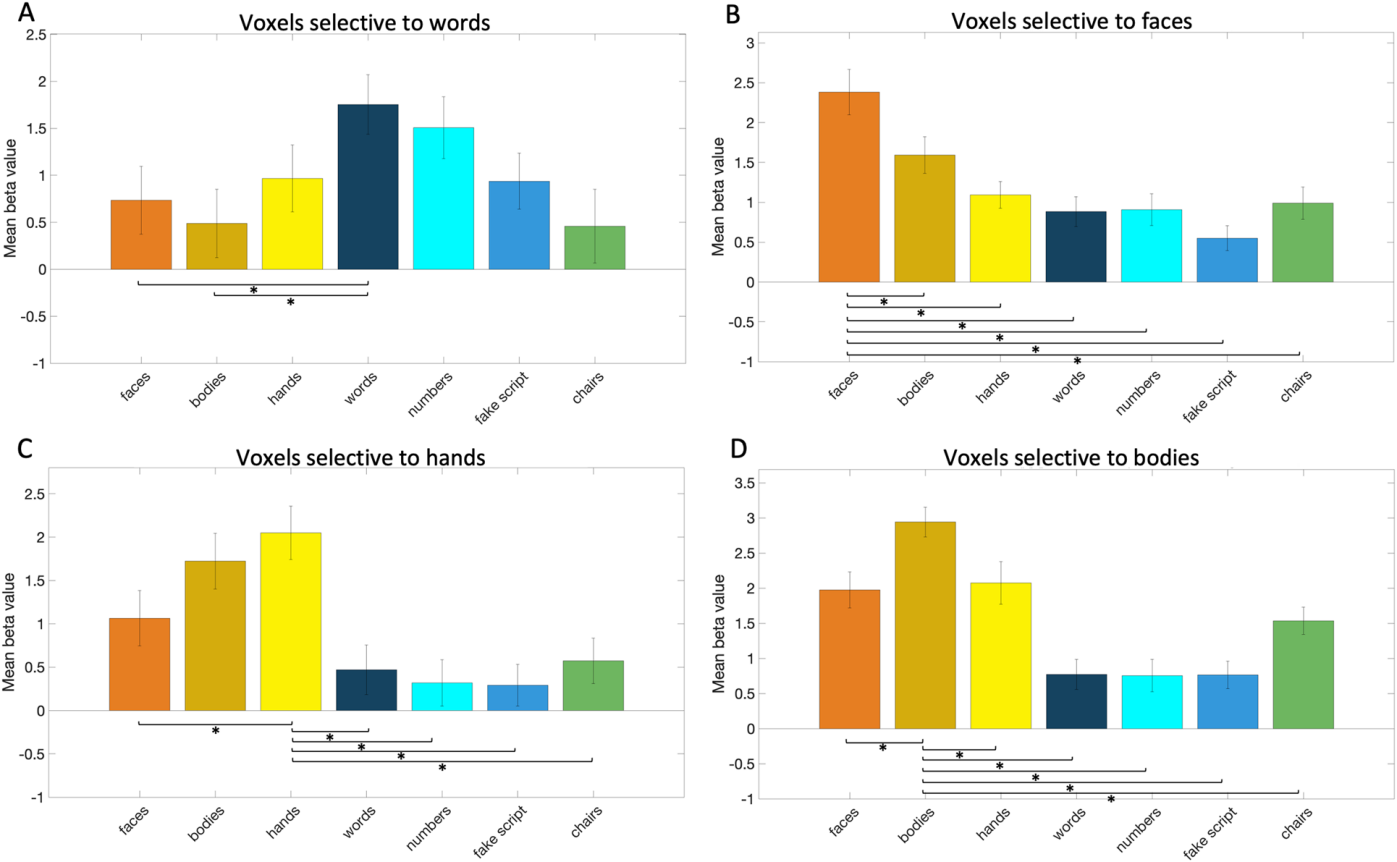
The response in word- (A), face- (B), hand- (C), and body- (D)selective voxels of the right hemisphere to seven categories (faces,bodies, hands, words, numbers, fake script, and objects: chairs).Responses were calculated using data that were independent from thedata used to select the voxels (see Methods). Error bars representthe standard error, and the lines and stars indicate which pairedt-tests between conditions were significant (*p*< .008).

Like in the left hemisphere, we could indicate three clusters of faceselectivity, suited to be defined as IOG, pFus, and mFus in most subjects.Regarding the IOG, all but one subject (subject 10) showed the IOG clusterof face selectivity. Some subjects (1, 3, 4, 5, 10, 14) did not show a pFusarea at all or the activity seemed more likely to be a part of the firstface selectivity cluster (IOG) and/or the third face selectivity cluster(mFus). Like in the left hemisphere, the separation from the third/mFus areawas not always clear (e.g., subject 7, 8, and 18). The third cluster ofselectivity to faces (mFus) was detected in most subjects (except subject10, 11, and 17). With the split-half analysis, we were able to identifyface-selective (in 11 subjects) voxels across the middle and anteriorventral surface (seeFig. 8B, forstatistical details seeSupplementary Table 1). Across these voxels, activity (asquantified by the beta value, see Methods section) to faces wassignificantly (i.e.,*p*< .008 based on Bonferronicorrection) higher than to any of the other main categories. Like the lefthemisphere, we also located the mid-fusiform sulcus in the right hemisphereand found similar results as in the left hemisphere.

Like in the left hemisphere, we often found two, but rarely three, areas ofselectivity to hands. The posterior hand area appeared in all the subjectsin the right hemisphere. The second cluster of selectivity to hands was alsoapparent in most of the subjects (except subject 4, 5, 6, 7, 10, and 11).The third cluster of selectivity to hands could not be determined in most ofthe subjects (only in subject 9, 11, and maybe 17). Also alike in the lefthemisphere, we could instead around this location find selectivity to bodiesin some subjects (subject 3, 12, 14, and 16). With the split-half analysis,we were able to identify (in 16 subjects) hand-selective voxels across themiddle and anterior ventral surface. Across these voxels, activity (asquantified by the beta value, see Methods section) to hands wassignificantly (i.e.,*p*< .008 based on Bonferronicorrection) higher than to faces, words, numbers, fake script, and objects(chairs). It was not significantly different from bodies (significant onlyat uncorrected threshold:*p*< .05) (seeFig. 8C, for statistical details seeSupplementary Table1). A big difference between the left and right hand areas wasthe strength of the selectivity for words. Indeed, the difference betweenhands and words was smaller in the left hand-selective voxels as compared tothe right hand-selective voxels (*t*(14) = -2.78,*p*= .02), suggesting that the left hand area ismore selective to words than the right hand area.

Regarding the body areas, we again found those separate from the hand areason the ventral surface. We identified (in 14 subjects) body-selective voxelsacross the middle and anterior ventral surface using the split-halfanalysis. We found that activity (as quantified by the beta value, seeMethods section) to bodies was significantly (i.e.,*p*< .008 based on Bonferroni correction) higher than to faces and tohands, in contrast to the left hemisphere body-selective voxels. Theactivity to bodies was also significantly higher than to words, numbers,fake script, and objects (chairs) (seeFig.8D, for statistical details seeSupplementary Table2).

With the split-half analysis, we could also identify voxels selective tonumbers and to fake script across the middle and anterior ventral surface,but there was no evidence for more selectivity to numbers than words or tofake script than words, in the number and fake script areas. First, acrossthese number-selective voxels (identified in 14 subjects), activity (asquantified by the beta value, see Methods section) to numbers wassignificantly (i.e.,*p*< .008 based on Bonferronicorrection) higher than to faces, bodies, hands, fake script, and objects(seeSupplementary Fig.4A, for statistical details seeSupplementary Table2). It was not significantly different from words. Second, acrossthese fake script-selective voxels (identified in 11 subjects), activity (asquantified by the beta value, see Methods section) to fake script wassignificantly (i.e.,*p*< .008 based on Bonferronicorrection) higher than to faces, bodies, hands, and objects (seeSupplementary Fig.4B, for statistical details seeSupplementary Table2). It did not differ significantly from words and from numbers.Lastly, given that on the map of the ventral surface, we used selectivity toa typical objects category (chairs) as a reference landmark, we alsoattempted to identify object-selective voxels using the split-half analysis.Across these object-selective voxels (identified in 15 subjects), activity(as quantified by the beta value, see Methods section) to objects wassignificantly (i.e.,*p*< .008 based on Bonferronicorrection) higher than to faces and to numbers, but did not differsignificantly (significant only at uncorrected threshold:*p*< .05) from hands, words, and fake script. It was also not differentfrom bodies (seeSupplementary Fig. 4C, for statistical details seeSupplementary Table2).

##### 3.1.3.1 The location of hand selectivity cannot serve as a reference point to locate word and face selectivity in the right hemisphere

Then, we compared the locations of these areas in the right hemisphere tothe locations in the left hemisphere and investigated if the hand areasin this hemisphere could also consistently locate the VWFA subareas andthe typical face areas, like in the left hemisphere. In conclusion, thehand areas could not serve as a reference landmark like in the lefthemisphere, in part due to the more complex nature of the hand and wordselectivity in the right hemisphere. For more details, we refer to theSupplementary Results.

#### 3.1.4 The organization of word- and other category-selective areas in left-handed subjects

Three subjects in our subject pool were left-handed: subject 2, 7, and 17. Werefer toSupplementaryFigure 2for all results described below. Looking at the lefthemisphere, subject 2 and subject 7 showed the same organization in theirleft hemisphere as the right-handed subjects did, as described above.However, subject 17 differed from this. In the left hemisphere, we found thefirst hand cluster, the second word cluster anterior to that, the pFuscluster medial to that, and the second hand cluster below it. An mFus areaalso seemed to be present in this subject 17. All the other clusters weremissing: the first (unless very small and distributed) and third wordcluster, the IOG cluster, and the third hand cluster.

Looking at the right hemisphere of the left-handed subjects, in subject 17,we could find an organization of areas somewhat like the one found in theleft hemisphere of our right-handed subjects. The subject showed all firstand second hand, word, and face areas. In the right hemisphere of subject 2,we found the standard organization of selectivity like that of the lefthemisphere in the right-handed subjects. We found the first hand cluster,the first word cluster, the IOG cluster, the second hand cluster, the secondword cluster, pFus, and mFus. This means that we did not clearly see a thirdcluster of selectivity to words. The right hemisphere organization ofselectivity of subject 7 seemed similar but less clear than that of subject2.

### 3.2 Character categories group together in representational space

To achieve a mapping of word selectivity relative to other visual categories, weinvestigated the data in two ways: the functional neuroanatomy and therepresentational space. For every subject, two different matrices were createdthrough MVPA: for the OTC ROI, separately for the left (average number ofvoxels: 3776.37, standard deviation: 795.02) and the right (average number ofvoxels: 2894.26, standard deviation: 848.64) hemisphere. Each of these matriceswere normalized and then averaged across all subjects. These matrices can befound inSupplementaryFigure 5. These matrices were consistent: every subject’smatrix was significantly correlated with the average matrix (mean correlationfor the left OTC ROI:*r*= .80, mean correlation for theright OTC ROI:*r*= .74).

These matrices were then visualized in a two-dimensional space using MDS. Wecalled this result the representational space of our categories (seeFig. 9). Within the visualization of thematrices in a 2D space, categories that were located close together shared amore similar neural representation than categories that were further away fromeach other. Inside this space, we also visualized the Procrustes-transformed MDSresults of every participant for each category using one line per participantand found that overall, our subjects presented with consistent results. Welooked first at the results from the left OTC ROI. We noted some generalfindings: a grouping of character categories (words, numbers, fake script), agrouping of animate categories (faces, bodies, cats, hands), and a group ofinanimate categories in the middle of the plot with the following categories:cars, fish, chairs, instruments, flowers, vegetables, hammers, scissors,buildings, trees, and cubies. Fish are animate shapes but in MVPA results theyappear close to inanimate categories (Connollyet. al, 2012). Cubies and smoothies were unknown shapes for theparticipants. The cubies fell with the inanimate group, whereas the smoothiescould be included within the animate group in the plot. This might be explainedby differences in mid-level features between the two categories, based onprevious research results: animate categories have a higher curvilinearity thanartifacts (Levin et al., 2001), perceivedcurvature predicts the classification of animacy of a texform, and the curvaturedifferences between animals and artifacts can explain to some degree themixed-animacy search advantage (Long et al.,2017). We found that the control condition (scrambled) was locatedseparately from all other categories. When looking at the right OTC ROI, wefound similar results.

**Fig. 9.**
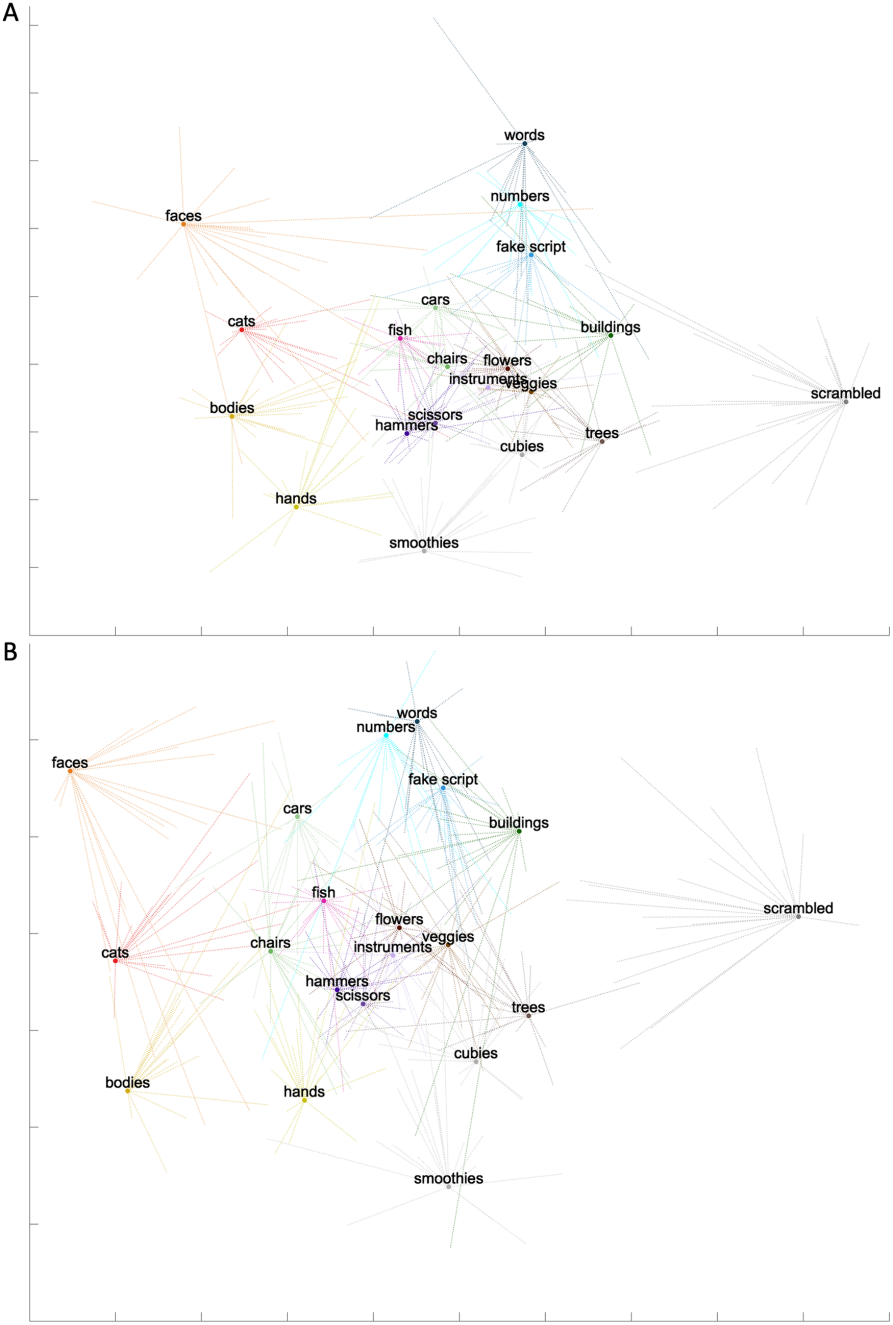
Visualization of the average MVPA matrix of the (A) left OTC ROI and (B)right OTC ROI, using MDS. MDS was also applied on each individual MVPAmatrix, and these MDS results were then Procrustes transformed to theaverage MDS result. The individuals’ Procrustes-transformed MDSresults are depicted by one line per subject for every category withinthe space.

Then, we focused on understanding the position of words and other charactercategories in particular, inside this MDS space. Characters were groupedtogether. Using paired t-tests (i.e.,*p*< .006 based onBonferroni correction), we investigated if numbers and fake script indeed weresignificantly more similar to words than other seemingly close categories likecars and faces. The distance (based on the normalized LDC value of everysubject) between words and numbers was significantly smaller than the distancebetween words and cars in both the left (*t*(18) = -10.81,*p*= 2.64*10^-9^) and the righthemisphere (*t*(18) = -5.84,*p*=3.32*10^-5^). The same was true when comparing the distancebetween words and numbers with the distance between words and faces in both theleft (*t*(18) = -11.06,*p*=1.86*10^-9^) and the right hemisphere(*t*(18) = -12.92,*p*=1.53*10^-10^). To conclude, numbers were significantly moresimilar in its neural activation pattern to that of words, as compared to othercategories that seemed the closest in the MDS space. We then investigated thesame for the other character category: fake script. The distance between wordsand fake script was significantly smaller than the distance between words andcars in both the left (*t*(18) = -7.07,*p*= 1.37*10^-6^) and the right hemisphere(*t*(18) = -4.18,*p*= .0006).The same was true when comparing the distance between words and fake script withthe distance between words and faces in both the left (*t*(18)= -10.09,*p*= 7.82*10^-9^) andthe right hemisphere (*t*(18) = -12.26,*p*= 3.55*10^-10^). As a conclusion, both numbers and fakescript were significantly closer to words than other categories. Charactercategories thus grouped together in the representational space of the OTC.

Second, we explored if faces, compared to other relevant human-relatedcategories, showed a special relation to the position of words in therepresentational space of the OTC, given that these categories have beenpreviously implicated to compete for cortical territory during development. Weperformed paired t-tests (i.e.,*p*< .01 based onBonferroni correction) to investigate if the neural activation pattern of faceswas more similar to that of words than bodies and hands. InFigure 9, we observed that bodies and hands were furtheraway than faces. The distance between words and faces was not significantlysmaller than the distance between words and bodies in the left(*t*(18) = -1.18,*p*= .25)hemisphere, but it was significantly smaller in the right hemisphere(*t*(18) = -4.31,*p*= .0004).When comparing the distance between words and faces to the distance betweenwords and hands, we found that the distance between words and faces was notsignificantly smaller than the distance between words and hands in the lefthemisphere (*t*(18) = -0.38,*p*=.71), nor in the right hemisphere (*t*(18) = 1.63,*p*= .12). To summarize, in the left hemisphere,faces was not the closest category to words: bodies/hands were as close to wordsas faces was. In the right hemisphere, faces was also not the closest categoryto words: faces was closer to words than the bodies, but hands was as close towords as faces was. This suggested that in the representational space, nospecial relationship existed between words and faces specifically, compared toother human-related categories.

Third, we sought out the closest inanimate category (cars) in the MDS space andcompared its distance to words with what seemed to be the closest/mostrepresentationally similar animate category in the MDS space (faces) to words.The distance between words and faces was not significantly different from thedistance between words and cars (left hemisphere:*t*(18)= 3.94,*p*= .22; right hemisphere:*t*(18) = 6.07,*p*= .15). Thissuggested that faces, compared to another seemingly close inanimate category,was not more or less similar in terms of the neural activity pattern. This wasfurther evidence against a special relationship between words and faces in therepresentational space of the OTC.

Finally, we compared the left to the right hemisphere. When looking at thefunctional neuroanatomy of the ventral OTC, we found that the right hemisphere,as compared to the left hemisphere, showed a weaker selectivity to words.Word-selective areas, identified using the split-half analysis, were notsignificantly more responsive to words than to other types of characters in theright hemisphere. In addition, in most of the subjects, we could not localizethe third/anterior cluster of word selectivity on the right ventral brainsurface. On the other hand, the organization in the representational space ofthe left and right hemisphere (Fig. 9A, B)seemed very similar. We sought to understand if the left and right hemisphereprocessed words and other characters in a similar way in terms ofrepresentational similarity. To this end, we compared the normalized LDC valueof each subject for several pairs of categories between the left and righthemisphere: for words versus numbers and for words versus fake script. We foundthat one of the two tests was almost significant (i.e.,*p*< .03 based on Bonferroni correction): words versus numbers(*t*(18) = 2.25,*p*= .04). Theother test, words versus fake script, was significant (*t*(18)= 2.53,*p*= .02). Thus, the neural pattern ofwords differed more from the pattern of numbers and fake script in the lefthemisphere than in the right hemisphere. This was in accordance with the resultsfrom the split-half analysis, where the left hemisphere differentiated betweenwords and other characters, but the right hemisphere did not.

## 4 Discussion

In this study, we investigated where visual word forms are located on the ventralsurface map of the functional neuroanatomy of the occipitotemporal cortex (OTC). Werelated these findings to other category-selective areas implicated by the neuralrecycling theories and the proposal byYeatman etal. (2021, see Introduction) (other character categories: numbers andfake script, and human-related categories: faces, hands, and bodies). In addition,we also explored word selectivity in the representational space and if there was aspecial relationship between words and faces (and in extension possibly also handsand bodies) here. We scanned 19 participants with 7T fMRI and presented them withshapes of 20 different categories. Our analyses were conducted at the level of theindividual brain (thus, without spatial normalizing or smoothing).

We mapped the functional neuroanatomy of OTC by visualizing the selective activationto characters and human-related categories on the ventral brain surface. We alsoassessed replicability and the strength of category selectivity through a split-halfanalysis. To provide a reference point, we included a typical objects category(chairs). We found abundant and wide-spread selectivity to the categories ofinterest across the ventral surface.

In the left hemisphere of ventral OTC, we consistently identified three clusters ofword selectivity across participants. One cluster was situated posteriorly to thefusiform gyrus within the inferior occipital gyrus, one more anterior in theposterior part of the fusiform gyrus and occipitotemporal sulcus, and one even moreanterior within the anterior fusiform gyrus and occipitotemporal sulcus. Inaddition, we found an extra cluster of word selectivity that we called the pFus wordarea because it was positioned adjacent to or between the pFus face selectivityarea. Comparing these word areas to recent functional subdivisions, we suggest thatthe middle/second and the anterior/third word area correspond with the pOTS/VWFA-1and mOTS/VWFA-2 respectively (Lerma-Usabiaga et al.,2018;White et al., 2019). Theposterior/first word area aligns with posterior regions, sometimes definedseparately from the VWFA, as these regions exhibit selectivity for letters withoutspecific preference for word formations (James etal., 2005;Strother et al., 2015;Vinckier et al., 2007;Wong et al., 2009;Yeatman et al., 2021). This is also supported by the observation ofactivity to not just words, but also fake script and numbers in this posterior partof ventral OTC, whereas more anterior the activity was more specific to words.Notably, the designation of the pFus word area as a subarea of the VWFA appearsnovel. Since previous research has predominantly subdivided the VWFA based onfunctional differences instead of anatomically determined separations, it could bethat the pFus word area could not be discriminated from pOTS/VWFA-1 and/or becausethese studies did not localize the separate face areas. A recent study byBoring et al. (2021)localized word and faceselectivity at high spatial resolution in each individual subject. Comparing ourfindings to theirs, we suggest that the pFus word area, along with the posterior,middle, and anterior word areas, could be defined in most of their subjects. Inabout 75% of their subjects, the authors observed selectivity to words medial toselectivity to faces along the mid-fusiform sulcus. We believe we can find (some)evidence for this in a large part of our participants (2, 3, 5, 6, 7, 8, 9, 10, 11,12, 13, 15, 16) and this medial selectivity could often be linked (but was notnecessarily limited) to what we have termed the pFus word area (2, 3, 5, 6, 7, 8, 9,11, 12, 16). Word selectivity within the left hemisphere was confirmed through thesplit-half analysis, showing robust selectivity to words in the identified voxels.This selectivity was significantly higher compared to all other categories ofinterest, particularly evident when contrasting words with non-character categories.While some selectivity to other character-related categories (numbers and fakescript) was observed in the word-selective areas, it was notably weaker than theselectivity observed for words.

Considering the importance of the mid-fusiform sulcus in identifying face-selectiveregions (Weiner et al., 2014), we examinedits relevance to the word-selective areas in the left hemisphere of OTC. While itdid not play a critical role in locating these word areas, some associations withthe word areas were noted. The split-half analysis replicated the face selectivity,demonstrating stronger selectivity to faces in these areas compared to othercategories of interest.

Surprisingly, in the left hemisphere, we also identified at least two clusters ofhand selectivity: one lateral and posterior (within inferior occipital gyrus andlateral occipital sulcus), which has been demonstrated before byBracci et al. (2010), and one more in the middle of theventral surface of OTC (along the middle of the fusiform gyrus, sometimes includingthe occipitotemporal sulcus). These hand-selective regions exhibited robustselectivity to hands, as confirmed by the split-half analysis, where hand activitysurpassed that of all other categories of interest. Interestingly, the difference inactivity between hands and words, faces, and bodies was smaller than between handsand objects, numbers, and fake script. This unexpected observation (since words areinanimate shapes, like the other character categories and objects) suggests apotential special connection between words/letters and hands, possibly due to theirfrequent association (both in a visual and motoric sense) during typing, writing,and reading tasks. This aligns with recent research byNordt et al. (2021), indicating a competitiverelationship between limb and word selectivity. Additionally, we observedbody-selective regions in the ventral left hemisphere, with the split-half analysisconfirming their replicability and strong selectivity to hands and faces, albeit notsignificantly stronger than to bodies. An extra test suggested a trend that thehand-selective areas showed a more distinct selectivity to its preferred category(hands) than the body-selective areas did to bodies.

Remarkably, the hand-selective regions served as reliable landmarks for locatingword- and face-selective areas on the left hemisphere ventral surface of OTC. Medialto the first hand was the first word and face area in the inferior occipital gyrus.The second hand area appeared more anterior than the first one around the middle ofthe surface along fusiform gyrus and occipitotemporal sulcus. In between this firstand second hand area, the second word area was located and more medial to that, thepFus face area, which was joined or broken up by what we called the pFus word area.In some of the subjects, there was also a third hand area even more anterior on theventral OTC surface. Below/around the second hand area and in between this secondand the third hand area, we found the mFus face area and the third word area (withinanterior fusiform gyrus and occipitotemporal sulcus). These findings align with theproposal byYeatman et al. (2021)(which wasin part based onGrill-Spector & Weiner,2014), suggesting that VWFA-1 and VWFA-2 are positioned adjacent to thepFus and mFus face areas, with a body/limb area subdividing VWFA-1 and VWFA-2.Interestingly, we observed that the hand areas, more so than the body areas,provided guidance for the subdivisions of word (and face) areas. Moreover, weidentified not just one hand area between VWFA-1 and VWFA-2, but two (or eventhree), contributing to the subdivision between the first/posterior word area andVWFA-1 (second/middle word area).

Regarding the right ventral surface of OTC, we could consistently identify the firstand often also the second word-selective cluster. We could not identify thethird/anterior word area, and this aligns withWhiteet al. (2019)that could only define the VWFA-2 in the right hemispherein a minority of their subjects. Unlike the left hemisphere, selectivity withinthese right hemisphere word areas leaned more towards characters in general(including numbers and fake script) rather than specifically to words. Thesplit-half analysis confirmed this result, revealing that while word-selectivevoxels were identifiable, their activity to words did not significantly surpass thatto other character categories, consistent with prior research (Dehaene et al., 2004;Vinckier et al., 2007). Given their response to various letter forms,naming these areas “word-selective” may be debatable. We could rarelyidentify the third word area, nor could we identify the pFus word area. Like in theleft hemisphere, the mid-fusiform sulcus did not play a critical role in locatingthe word areas, but did show certain relations to the word areas. We replicated faceselectivity with the split-half analysis. We could define the first and often alsothe second hand area and this selectivity was replicated in the split-half analysis.Interestingly, left hand-selective voxels showed greater selectivity to wordscompared to right hand-selective voxels. Distinct body areas were found separatefrom the hand areas, with the split-half analysis revealing their stronger activityto bodies compared to faces and hands, unlike the left hemisphere body area. Due tothe more complex nature and location of word and hand selectivity in the righthemisphere, the hand areas could not serve as reliable reference points for locatingword and face selectivity. All these findings from the right hemisphere areconsistent with the idea of the left hemisphere being language dominant. Notably,among the three left-handed participants, two showed organization similar toright-handed participants in their left hemisphere, while the others displayedtypical left hemisphere organization in their right hemisphere.

In the representational space of both the left and right hemisphere OTC, charactercategories formed a distinct group separate from both animate and inanimatecategories. Numbers and fake scripts were notably closer to words than to faces orcars. We examined if faces were significantly closer to words than otherhuman-related categories, aiming to discern any special relationship between wordsand faces, as one might expect based on the observed competition for territorybetween these categories during development, as proposed by the neuronal recyclingtheory. However, faces did not exhibit significantly closer proximity to wordscompared to bodies and hands. Furthermore, results showed that faces did not liesignificantly closer to words than cars (which was the closest inanimate category towords in the plot). This suggests that, although these categories may compete duringdevelopment for cortical territory, within the representational space of theoccipitotemporal cortex there is no special relationship between words and faces.Finally, the visualization of representational space revealed a similar organizationin both hemispheres. However, further analysis showed that the neural patterns forwords and other categories were more similar in the right hemisphere than in theleft hemisphere, consistent with functional neuroanatomy findings in ventralOTC.

The word forms that were used in this study (akin to the numbers and fake scriptcategories) were strings of letters, which consisted of a string of letters of anunknown alphabet and of digits. In our split-half analysis results, word-selectiveareas in the left hemisphere exhibited significantly higher selectivity to wordscompared to numbers and fake script, suggesting differentiation between letters ofthe roman alphabet and other characters. Our letter strings were not pseudowords(all except one did contain at least one vowel and all were pronounceable) and wedid not include any real words (no semantic meaning). Importantly, in the past, wordselectivity has been localized with a variety of different contrasts (for a detailedoverview, seeCaffarra et al., 2021). Forexample, studies have differentiated between words, pseudowords, letter strings, andindividual letters (e.g.,Vinckier et al.,2007), whereas others have compared text in general to false fonts oreven non-linguistic categories such as faces (e.g.,Cohen et al., 2002;Dehaene et al.,2010). Only studies that differentiate between several categories likewords can isolate lexical-sensitive areas (Caffarraet al., 2021).Lerma-Usabiaga et al.(2018)confirmed this: only specific types of contrast could isolateanterior from posterior word selectivity and vice versa. However, they did find thatwith more general contrasts (such as word-fixation) they could activate bothposterior and anterior word selectivity, but of course could not make any functionaldistinctions between subregions in this way. In our study, we identified the VWFA bya contrast between letter strings and numerous other categories (some word-like andmany not word-like categories). We then divided the VWFA into subregions based onclustering we found within the single-subject high-resolution anatomy of the ventralOTC. We could not make functional distinctions between the found subregions due to alack of word-like categories and thus different contrast types. Another limitationof this study pertains to the results of the functional neuroanatomy, morespecifically the organization of the areas that we identified. Across all subjects,we could deduce a standard organization of word, face, and hand areas in the lefthemisphere, but not every subject showed this so clearly. For example, threesubjects did not show the third word area in the left hemisphere. This complicatedthe process of identifying and localizing areas.Supplementary Figure 1contains an overview of areas found per subject. However, small deviations from astandard organization are to be expected based on individual variability. While weobserved this consistent pattern of different category-selective areas in relationto each other, we could not describe any consistently predictive anatomicallandmarks across participants (nonetheless, the mid-fusiform sulcus was described),as the variability of the anatomical location of the word areas was too high. Thisis an important limitation of our study, given that such a finding would havefurther improved the reliability of localizing the word areas in this study and forfuture studies. Based on our study, it remains important to include other categoriesthan just words to localize the word areas.

This study used 7T fMRI, offering several advantages. First, it provided data of highquality and spatial resolution, as evidenced, for example, by the observation thatin volume-space, activity was nicely restricted to grey matter (seeFigures 6and7). Second, it allowed us to work at the level of the individual,without spatial normalization or smoothing of the data, preserving unique gyral andsulcal patterns of each brain and thus allowing a more accurate localization ofactivity (Weiner & Grill-Spector,2013), which was a main aim of this study. Spatial smoothing can also lead toaveraging together of regions that actually lie distant from each other on thesurface (Weiner & Grill-Spector,2013), which would have also been detrimental to the aim of our study. Groupaveraging can also have detrimental effects: it lacks spatial preciseness(especially when areas vary in size and/or location between subjects) and boundariesbetween areas can get mixed (Glezer &Riesenhuber, 2013;Wandell et al.,2012). Following recommendations byBrodoehl et al. (2020), our analysis pipeline employed a general linearmodel analysis projected onto individual brain surfaces without normalization orspatial smoothing. This approach aligns with recent calls to investigate the VWFA atthe individual level (Caffarra et al., 2021;Yeatman et al., 2021). Third, the use of7T allowed us to present each subject in just one scan session with many differentcategories, enabling a more holistic view on the functional organization of theOTC.

This study represents, to our knowledge, the first investigation of high spatialresolution mapping of word selectivity and the delineation of its subareas,alongside other potentially relevant categories. BothCaffarra et al. (2021)andYeatman et al. (2021)recently emphasized the importanceof investigating where precisely, at the level of the individual brain, wordselectivity was located in OTC and the potential existence of anatomically divisiblesubareas of word selectivity. Across participants, we consistently identified threesubareas of word selectivity and an additional area termed the pFus word area on theventral surface of the OTC in the left hemisphere. These findings provide afoundation for future studies to consistently define subregions of the visual wordform area (VWFA). Furthermore, by relating these word areas to other areas ofselectivity, we discovered hand areas that were instrumental in locating these wordareas and the well-known face areas (IOG, pFus and mFus), offering an avenue forfuture studies to incorporate hand categories in localizing VWFA subareas. We alsoobserved that other categories than just faces may be relevant to the competitionwith words for cortical territory, given that the hand areas provided a referencelandmark for the word areas. This aligns with the results found byNordt et al. (2021). Additionally, our investigationexplored the potential special relationship between words and faces within therepresentational space of the OTC. These categories compete for cortical territoryduring development according to the neuronal recycling theory (Dehaene & Cohen, 2007;Dehaene et al., 2010). In the representational space, wefound no evidence for a special relationship between words and faces.

Future research could explore differences in functional connectivity between subareasof the VWFA and other brain regions. While studies have examined the anatomicalconnectivity of the VWFA (for an overview, seeCaffarra et al., 2021;Yeatman et al.,2021), investigating the functional connectivity of VWFA subareas couldprovide novel insights. Furthermore, it would be valuable to investigate potentialfunctional differences among the subareas observed in this study. For example, aprocessing gradient may exist from the posterior to the anterior subareas of theVWFA. Based on many different studies (e.g.,Lerma-Usabiaga et al., 2018;Vinckier etal., 2007;White et al., 2019;Woolnough et al., 2020),Caffarra et al. (2021)proposed a posterior to anteriorprocessing model, suggesting that more posterior regions represent perceptualinformation of written language while more anterior portions are sensitive tolinguistic aspects of words. The authors also considered the temporal dynamics andanatomical connectivity found in left ventral OTC in this word processing model.Additionally, a similar study incorporating pseudowords (considering the naturalcharacteristics of language) and real words could explore functional distinctionsbetween VWFA subregions identified in this study, potentially augmenting ourunderstanding of the results observed in the representational space. Lastly, futureresearch on the VWFA should consider the inclusion of categories beyond faces, suchas hands, not only to explore the competition posited by neural recycling theories,but also to investigate the location and function of category-selectivesubareas.

## Supplementary Material

Supplementary Material

## Data Availability

All de-identified data are available fromhttps://doi.org/10.12751/g-node.96eqnl. Following the privacy laws ofGDPR, the raw (f)MRI data contain potentially identifying or sensitive participantinformation and are therefore only available upon request from the authors or fromthe research institute throughdatamanager@nin.knaw.nl.
