# Supplementary material for: The position of visual word forms in the anatomical and representational space of visual categories in occipitotemporal cortex
Source: Imaging Neurosci (Camb). 2024 Jun 25;2:imag-2-00196. doi: 10.1162/imag_a_00196 (PMC12272258; doi:10.1162/imag_a_00196)
Supplement: Supplementary Material [file imag_a_00196-supp.pdf]

**Supplementary Tables**

**Supplementary Table 1**

*Statistical details from paired t-tests in the word, face and hand areas of the left and right hemisphere*

| Paired *t*-tests | *t* | *p* | | *t* | *p* |
|---|---|---|---|---|---|
| Left word areas (*df*=14) | | | Right word areas (*df*=10) | | |
| Words-faces | 11.66 | $1.35*10^{-8}$ | Words-faces | 4.65 | .0009 |
| Words-hands | 12.63 | $4.84*10^{-9}$ | Words-hands | 2.88 | .02 |
| Words-bodies | 12 | $9.15*10^{-9}$ | Words-bodies | 5.06 | .0005 |
| Words-numbers | 7.81 | $1.81*10^{-6}$ | Words-numbers | 2.05 | .07 |
| Words-fake script | 11.64 | $1.38*10^{-8}$ | Words-fake script | 2.83 | .02 |
| Words-objects | 18.22 | $3.79*10^{-11}$ | Words-objects | 3.18 | .01 |
| Left face areas (*df*=13) | | | Right face areas (*df*=10) | | |
| Faces-words | 6.47 | $2.11*10^{-5}$ | Faces-words | 8.45 | $7.24*10^{-6}$ |
| Faces-hands | 6.77 | $1.32*10^{-5}$ | Faces-hands | 6.74 | $5.08*10^{-5}$ |
| Faces-bodies | 12 | $2.07*10^{-8}$ | Faces-bodies | 6.55 | $6.46*10^{-5}$ |
| Faces-numbers | 9.16 | $4.91*10^{-7}$ | Faces-numbers | 11.24 | $5.38*10^{-7}$ |
| Faces-fake script | 9.98 | $1.85*10^{-7}$ | Faces-fake script | 8.61 | $6.13*10^{-6}$ |
| Faces-objects | 8.98 | $6.19*10^{-7}$ | Faces-objects | 9.76 | $1.99*10^{-6}$ |
| Left hand areas (*df*=15) | | | Right hand areas (*df*=15) | | |
| Hands-words | 3.64 | .002 | Hands-words | 7.52 | $1.83*10^{-6}$ |
| Hands-faces | 3.3 | .005 | Hands-faces | 4.5 | .0004 |
| Hands-bodies | 3.85 | .002 | Hands-bodies | 2.35 | .03 |
| Hands-numbers | 8.19 | $6.47*10^{-7}$ | Hands-numbers | 7.20 | $3.06*10^{-6}$ |
| Hands-fake script | 9.58 | $8.78*10^{-8}$ | Hands-fake script | 8.52 | $3.92*10^{-7}$ |
| Hands-objects | 6.3 | $1.43*10^{-5}$ | Hands-objects | 8.96 | $2.07*10^{-7}$ |

*Note*. *P*-values < .008 (Bonferroni correction) are significant.

**Supplementary Table 2**

*Statistical details from paired t-tests in the body, number, fake script and object (chair) areas of the left and right hemisphere*

| Paired *t*-tests | *t* | *p* | | *t* | *p* |
|---|---|---|---|---|---|
| Left body areas (*df*=14) | | | Right body areas (*df*=13) | | |
| Bodies-words | 6.31 | $1.93*10^{-5}$ | Bodies-words | 10.19 | $1.44*10^{-7}$ |
| Bodies-faces | 2.89 | .01 | Bodies-faces | 6.73 | $1.42*10^{-5}$ |
| Bodies-hands | 1.51 | .15 | Bodies-hands | 4.31 | .0009 |
| Bodies-numbers | 5.84 | $4.29*10^{-5}$ | Bodies-numbers | 9.28 | $4.27*10^{-7}$ |
| Bodies-fake script | 7.51 | $2.84*10^{-6}$ | Bodies-fake script | 10.5 | $1.01*10^{-7}$ |
| Bodies-objects | 7.41 | $3.32*10^{-6}$ | Bodies-objects | 10.13 | $1.55*10^{-7}$ |
| Left number areas (*df*=16) | | | Right number areas (*df*=13) | | |
| Numbers-words | 1.22 | .24 | Numbers-words | -0.37 | .72 |
| Numbers-faces | 8.27 | $3.57*10^{-7}$ | Numbers-faces | 5.09 | .0002 |
| Numbers-hands | 10.34 | $1.72*10^{-8}$ | Numbers-hands | 4.18 | .001 |
| Numbers-bodies | 10.15 | $2.23*10^{-8}$ | Numbers-bodies | 4.73 | .0004 |
| Numbers-fake script | 3.16 | .006 | Numbers-fake script | 4.31 | .0008 |
| Numbers-objects | 9.01 | $1.15*10^{-7}$ | Numbers-objects | 4.29 | .0009 |
| Left fake script areas (*df*=16) | | | Right fake script areas (*df*=10) | | |
| Fake script-words | -3.51 | .003 | Fake script-words | -1.73 | .11 |
| Fake script-faces | 4.77 | .0002 | Fake script-faces | 10.78 | $7.97*10^{-7}$ |
| Fake script-hands | 4.42 | .0004 | Fake script-hands | 5.61 | .0002 |
| Fake script-bodies | 5.27 | $7.60*10^{-5}$ | Fake script-bodies | 4.83 | .0007 |
| Fake script-numbers | -1.37 | .19 | Fake script-numbers | -0.56 | .59 |
| Fake script-objects | 3.57 | .003 | Fake script-objects | 3.41 | .007 |
| Left object areas (*df*=18) | | | Right object areas (*df*=14) | | |
| Objects-words | 3.38 | .003 | Objects-words | 2.69 | .02 |
| Objects-faces | 2.70 | .01 | Objects-faces | 3.13 | .007 |
| Objects-hands | 2.8 | .01 | Objects-hands | 2.29 | .04 |
| Objects-bodies | 2.75 | .01 | Objects-bodies | 0.95 | .36 |
| Objects-numbers | 4.38 | .0004 | Objects-numbers | 4.11 | .001 |
| Objects-fake script | 4.39 | .0004 | Objects-fake script | 2.66 | .02 |

*Note*. *P*-values < .008 (Bonferroni correction) are significant.

## Supplementary Figures

| Left hemisphere | Subject 1 | Subject 2 left-handed | Subject 3 | Subject 4 | Subject 5 | Subject 6 | Subject 7 left-handed | Subject 8 | Subject 9 | Subject 10 | Subject 11 | Subject 12 | Subject 13 | Subject 14 | Subject 15 | Subject 16 | Subject 17 left-handed | Subject 18 | Subject 19 | Total |
|---|---|---|---|---|---|---|---|---|---|---|---|---|---|---|---|---|---|---|---|---|
| **Posterior word** | Yes | Yes | Yes | Yes | Yes | Yes | Yes | Yes | Yes | Yes | Yes | Yes | Yes | Yes | Yes | Yes | No (unless very small and distributed) | Yes | Yes | 18 |
| **Posterior face** | Yes | Yes | Yes | Yes | Yes | Yes | Yes | Yes | Yes | Yes | Yes | Yes | Yes | Yes | No | Yes | No | Yes | Yes | 17 |
| **Posterior hand** | Yes | Yes | Yes | Yes | Yes | Yes | Yes | Yes | Yes | Yes | Yes | Yes | Yes | Yes | Yes | Yes | Yes | Yes | Yes | 19 |
| **Middle word** | Yes | Yes | Yes | Yes | Yes | Yes | Yes | Yes | Yes | Yes | Yes | Yes | Yes | Yes | Yes | Yes | Yes | Yes | Yes | 19 |
| **Middle face** | Yes | Yes | Yes | Yes | Yes | Yes | Yes | Yes | Yes | Yes | Yes | Yes | Yes | No | Yes | Yes | Yes | Yes | Yes | 18 |
| **Middle hand** | Yes | Yes | Yes | Yes | Yes | Yes | Yes | Yes | Yes | Yes | Yes | Yes | Yes | Yes | Yes | Yes | Yes | Yes | Yes | 19 |
| **Anterior word** | Yes | Yes | Yes | No | Yes | Yes | No | Yes | Yes | Yes | Yes | Yes | Yes | Yes | Yes | Yes | No | Yes | Yes | 16 |
| **Anterior face** | Yes | Yes | Yes | Yes | Yes | Yes | Yes | Yes | Yes | Yes | Yes | Yes | No (unless very anterior cluster) | Yes | Yes | Yes (seperation from anterior unclear) | Yes | Yes | Yes (seperation from anterior unclear) | 18 |
| **Anterior hand** | Yes | No | No (bodies) | No | No | No | No (bodies) | No (bodies) | Yes | Yes | No | No | Yes | No | No | No | No | No (bodies) | Yes | 5 |
| **More anterior** | Yes faces and hands | No | Yes faces | Yes faces and hands | Yes faces and words | Yes faces | No | No | Yes faces | Yes faces | No | Yes faces and words | Yes faces (unless this is actually mFus) and words | No | No | Yes faces | No | No | Yes faces | 11 |
| **Object region posterior** | Yes | No | Yes | No | No | Yes | No | No | Yes | No | Yes | Yes | No | No | No | No | No | Yes | No | 7 |
| **pFus word area** | Yes | Yes | Yes | Yes | Yes | Yes | Yes | Yes | Yes | No | Yes | Yes | No | No | Yes | Yes | No | No | No | 13 |

| Right hemisphere | Subject 1 | Subject 2 left-handed | Subject 3 | Subject 4 | Subject 5 | Subject 6 | Subject 7 left-handed | Subject 8 | Subject 9 | Subject 10 | Subject 11 | Subject 12 | Subject 13 | Subject 14 | Subject 15 | Subject 16 | Subject 17 left-handed | Subject 18 | Subject 19 | Total |
|---|---|---|---|---|---|---|---|---|---|---|---|---|---|---|---|---|---|---|---|---|
| **Posterior word** | Yes (mostly other characters) | Yes | Yes | Yes | Yes (mostly other characters) | Yes | Yes | Yes (mostly other characters) | Yes | Yes | Yes | Yes | Yes | Yes (mostly to other characters) | Yes | Yes (mostly to other characters) | Yes | Yes (mostly other characters) | Yes | 19 |
| **Posterior face** | Yes | Yes | Yes | Yes | Yes | Yes | Yes | Yes | Yes | No | Yes | Yes | Yes | Yes | Yes | Yes | Yes | Yes | Yes | 18 |
| **Posterior hand** | Yes (more anterior than expected) | Yes | Yes (more anterior than expected) | Yes | Yes | Yes | Yes (more anterior than expected) | Yes | Yes | Yes (more anterior than expected) | Yes (longer towards anterior than expected) | Yes | Yes | Yes | Yes | Yes | Yes | Yes (more anterior than expected) | Yes | 19 |
| **Middle word** | Yes (medial, mostly other characters) | Yes (medial, bit lateral, mostly other characters) | Yes (medial) | Yes | Yes (medial) | Yes (mostly other characters) | Yes | Yes (medial, lateral, mostly to other characters) | No | No | No | Yes | Yes | Yes (medial, mostly to other characters) | Yes (medial, lateral) | No (seems part of pFus word area) | Yes | No | No | 13 |
| **Middle face** | No | Yes | No | No | No | Yes | Yes | Yes | Yes | No | Yes | Yes | Yes | No | Yes | Yes | Yes | Yes | Yes | 13 |
| **Middle hand** | Yes (mostly bodies) | Yes | Yes | No | No | No | No | Yes | Yes | No | No | Yes | Yes | Yes | Yes | Yes | Yes | Yes | Yes | 13 |
| **Anterior word** | No | No (small numbers, also numbers lateral) | No | No | Yes | No | No | No (small numbers) | No (small numbers) | No | No | Yes | No | No | No | No | No | Yes | No | 3 |
| **Anterior face** | Yes | Yes | Yes | Yes | Yes | Yes | Yes | Yes | Yes | No | No | Yes | Yes | Yes | Yes | Yes | No | Yes | Yes | 16 |
| **Anterior hand** | No | No | No (bodies) | No | No | No | No | No | Yes | No | Yes | No (bodies) | No | No (bodies) | No | No (bodies) | Yes | No | No | 3 |
| **More anterior** | Yes faces (unless it's part of mFus) | No | Yes faces and bodies | Yes faces | Yes faces and words | Yes faces | No | No | Yes faces | No | No | Yes faces | No | Yes faces | No | Yes numbers | Yes words | Yes hands | Yes faces | 12 |
| **Object region posterior** | Yes | Yes | No | No | No | Yes | Yes | No | Yes | Yes | Yes | Yes | Yes | No | Yes | No | No | No | No | 10 |
| **pFus word area** | Unclear | Yes ? (numbers) | Unclear | No | Unclear | No | No | No | No | No | No | No | No | Unclear | No | Yes ? (numbers) | No | No | No | 2 |

*Supplementary Figure 1.* Overview of which areas are found in which subjects on the ventral left (top) and in the right (bottom) hemisphere surface. Areas that are discussed on the rows of the table: the posterior word area, the posterior face area, the posterior hand area, the middle word area, the middle face area, the middle hand area, the anterior word area, the anterior face area, the anterior hand area, more anterior areas, the object region that is sometimes found posterior, the pFus word area, the ITG body area, the OTS body area. The columns are the 19 subjects and the last column indicates the total of subjects in which this area of that row could be found.

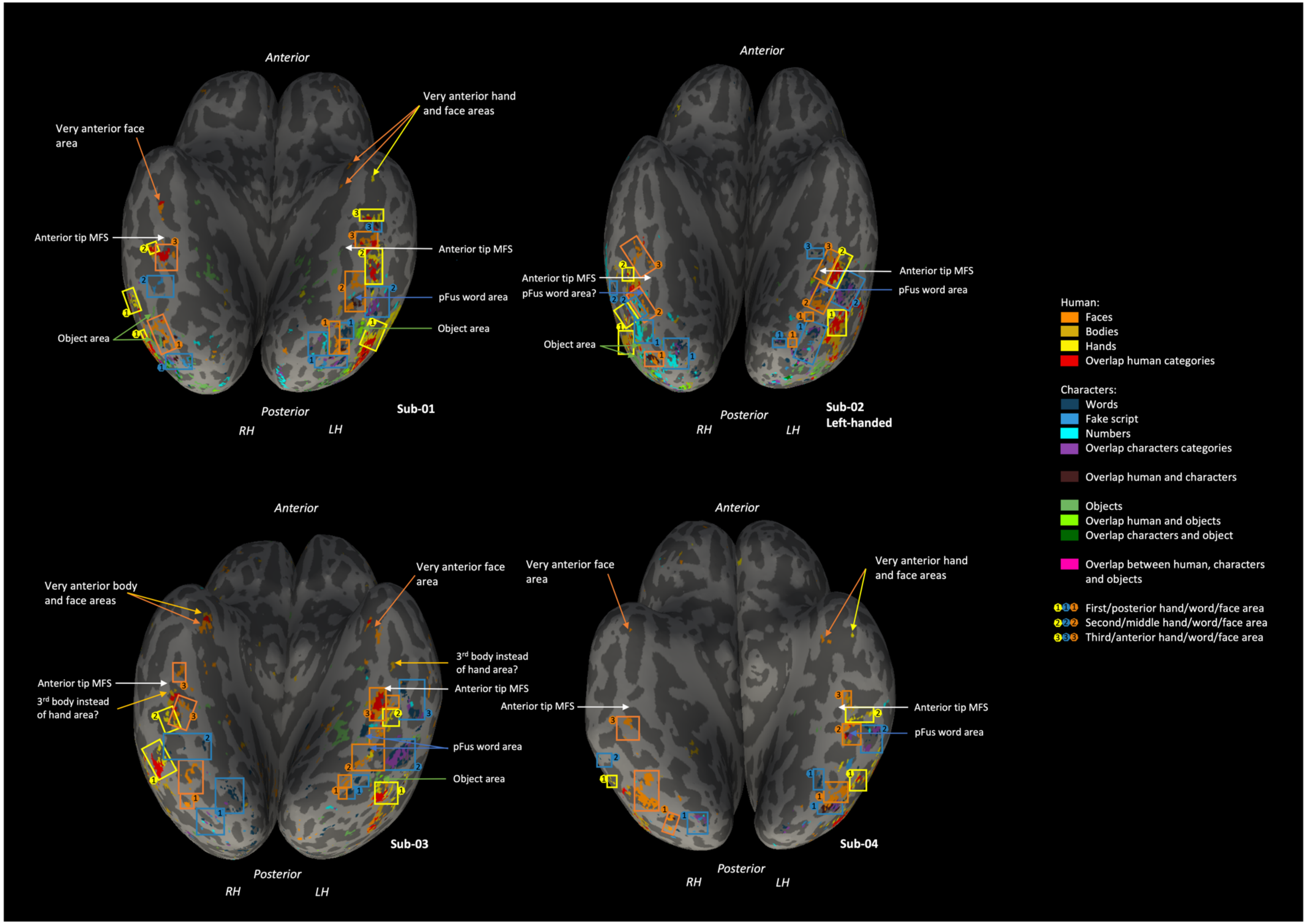
Anterior
Very anterior face area
Very anterior hand and face areas
Anterior tip MFS
Anterior tip MFS
pFus word area
Object area
Object area
Posterior
RH
LH
Sub-01
Anterior
Anterior tip MFS
pFus word area?
Object area
Anterior tip MFS
pFus word area
Posterior
RH
LH
Sub-02
Left-handed
Anterior
Very anterior body and face areas
Anterior tip MFS
3rd body instead of hand area?
Very anterior face area
3rd body instead of hand area?
Anterior tip MFS
pFus word area
Object area
Posterior
RH
LH
Sub-03
Anterior
Very anterior face area
Anterior tip MFS
Very anterior hand and face areas
Anterior tip MFS
pFus word area
Posterior
RH
LH
Sub-04
Human:
Faces
Bodies
Hands
Overlap human categories
Characters:
Words
Fake script
Numbers
Overlap characters categories
Overlap human and characters
Objects
Overlap human and objects
Overlap characters and object
Overlap between human, characters and objects
First/posterior hand/word/face area
Second/middle hand/word/face area
Third/anterior hand/word/face area

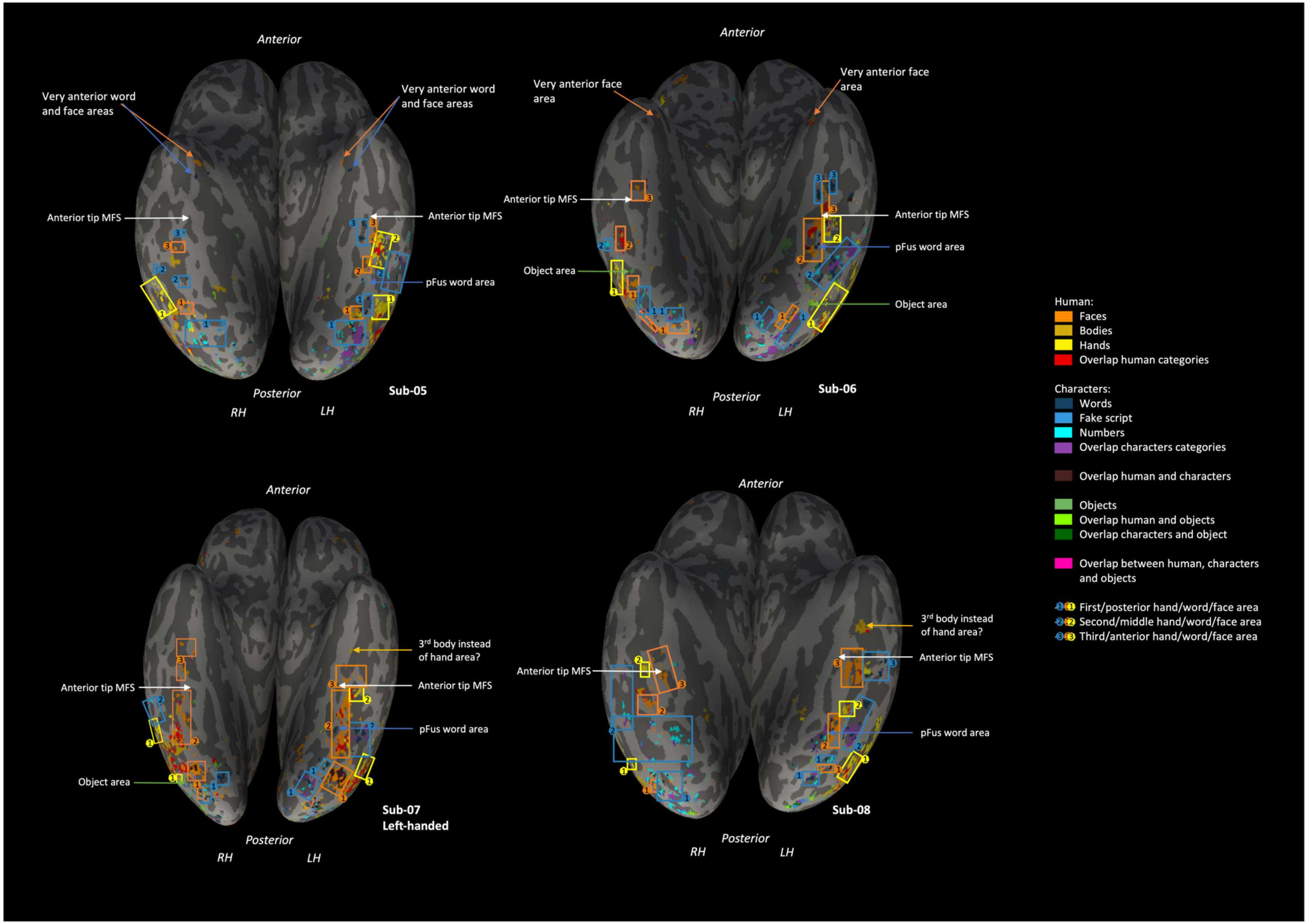
Anterior
Very anterior word and face areas
Very anterior word and face areas
Anterior tip MFS
Anterior tip MFS
pFus word area
Posterior
Sub-05
RH
LH
Anterior
Very anterior face area
Very anterior face area
Anterior tip MFS
Anterior tip MFS
pFus word area
Object area
Object area
Posterior
Sub-06
RH
LH
Anterior
3rd body instead of hand area?
Anterior tip MFS
Anterior tip MFS
pFus word area
Object area
Sub-07
Left-handed
Posterior
RH
LH
Anterior
3rd body instead of hand area?
Anterior tip MFS
Anterior tip MFS
pFus word area
Sub-08
Posterior
RH
LH
Human:
Faces
Bodies
Hands
Overlap human categories
Characters:
Words
Fake script
Numbers
Overlap characters categories
Overlap human and characters
Objects
Overlap human and objects
Overlap characters and object
Overlap between human, characters and objects
First/posterior hand/word/face area
Second/middle hand/word/face area
Third/anterior hand/word/face area

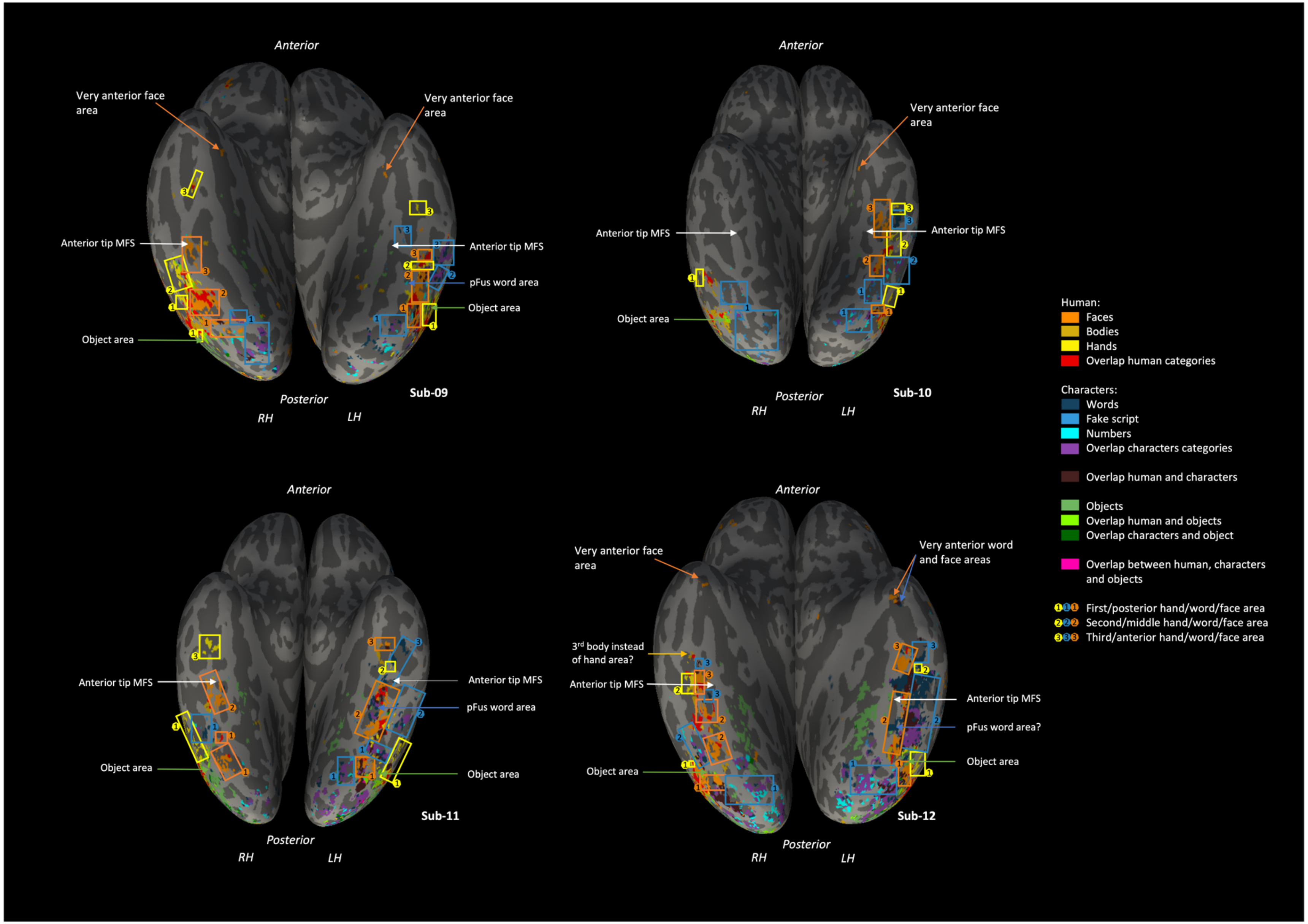

Anterior
Very anterior face area
Very anterior face area
Anterior tip MFS
Anterior tip MFS
pFus word area
Object area
Object area
Posterior
RH
LH
Sub-09
Anterior
Very anterior face area
Anterior tip MFS
Anterior tip MFS
Object area
Posterior
RH
LH
Sub-10
Anterior
Anterior tip MFS
Anterior tip MFS
pFus word area
Object area
Object area
Posterior
RH
LH
Sub-11
Anterior
Very anterior face area
Very anterior word and face areas
3rd body instead of hand area?
Anterior tip MFS
Anterior tip MFS
pFus word area?
Object area
Object area
Posterior
RH
LH
Sub-12
Human:
Faces
Bodies
Hands
Overlap human categories
Characters:
Words
Fake script
Numbers
Overlap characters categories
Overlap human and characters
Objects
Overlap human and objects
Overlap characters and object
Overlap between human, characters and objects
First/posterior hand/word/face area
Second/middle hand/word/face area
Third/anterior hand/word/face area

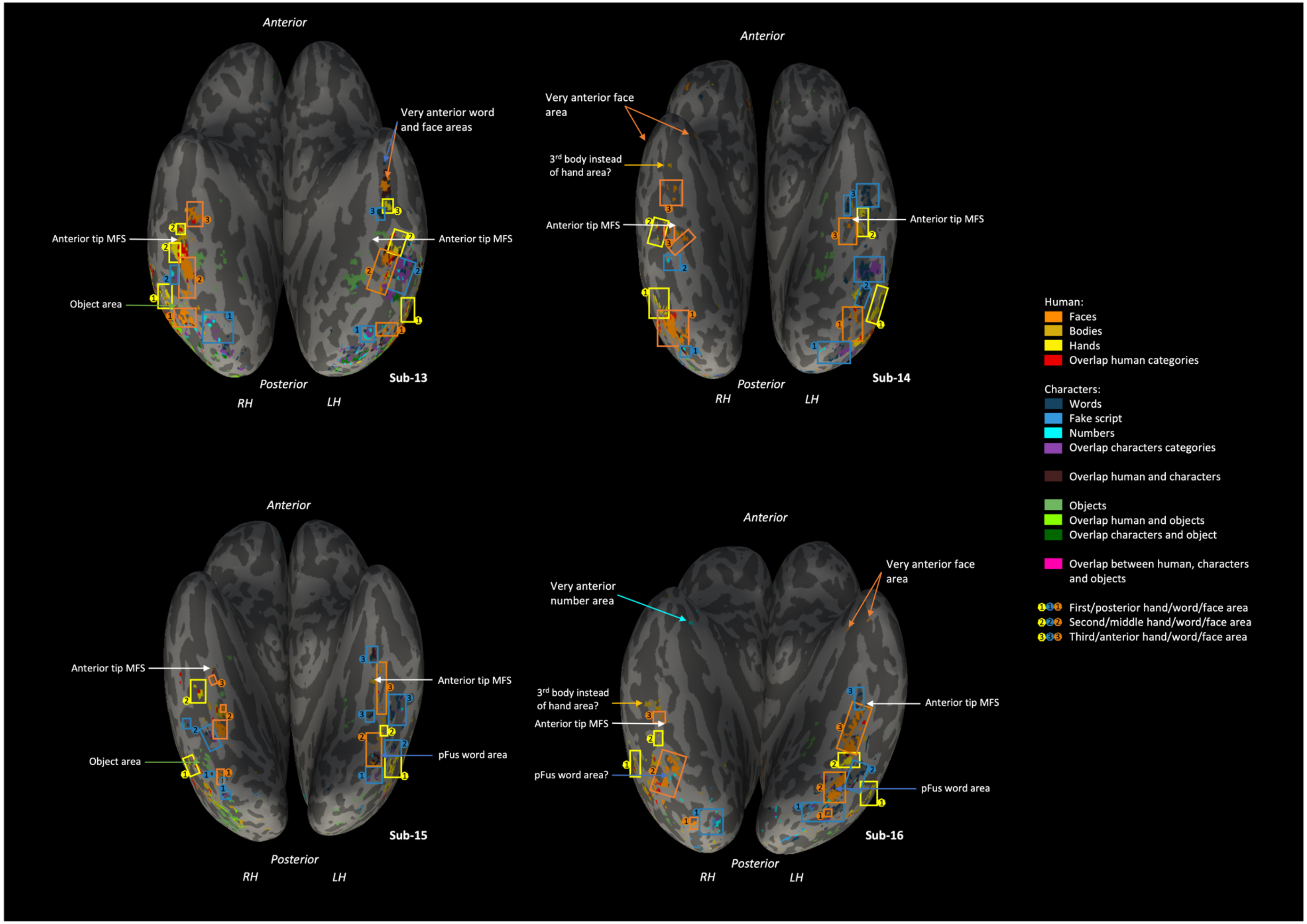

Anterior
Very anterior word and face areas
Anterior tip MFS
Anterior tip MFS
Object area
Posterior
Sub-13
RH
LH
Anterior
Very anterior face area
3rd body instead of hand area?
Anterior tip MFS
Anterior tip MFS
Posterior
Sub-14
RH
LH
Anterior
Anterior tip MFS
Anterior tip MFS
Object area
pFus word area
Posterior
Sub-15
RH
LH
Anterior
Very anterior number area
Very anterior face area
3rd body instead of hand area?
Anterior tip MFS
Anterior tip MFS
pFus word area?
pFus word area
Posterior
Sub-16
RH
LH
Human:
Faces
Bodies
Hands
Overlap human categories
Characters:
Words
Fake script
Numbers
Overlap characters categories
Overlap human and characters
Objects
Overlap human and objects
Overlap characters and object
Overlap between human, characters and objects
First/posterior hand/word/face area
Second/middle hand/word/face area
Third/anterior hand/word/face area

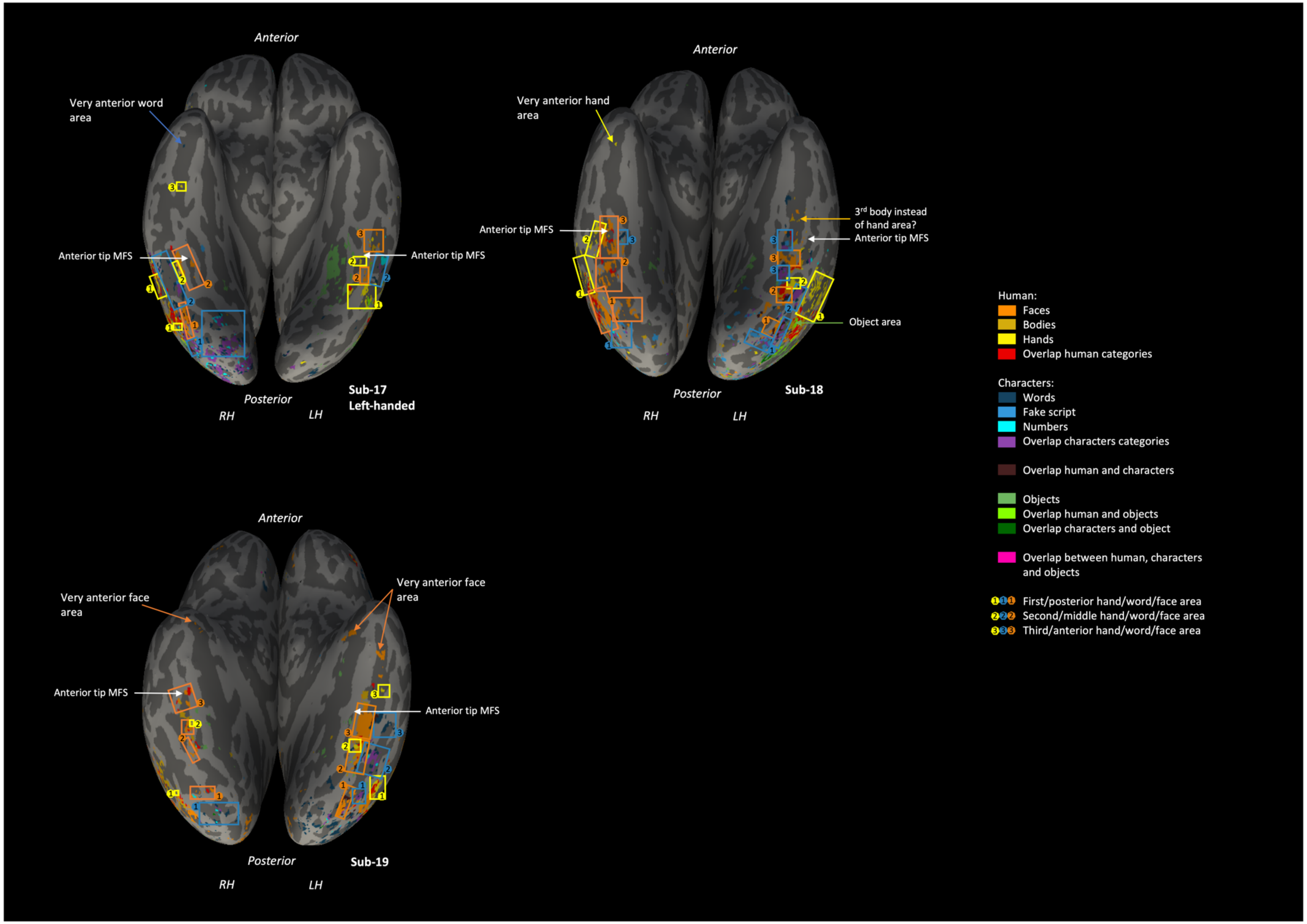

Anterior
Very anterior word area
Anterior tip MFS
Anterior tip MFS
Posterior
RH
LH
Sub-17
Left-handed
Anterior
Very anterior hand area
Anterior tip MFS
3rd body instead of hand area?
Anterior tip MFS
Object area
Posterior
RH
LH
Sub-18
Anterior
Very anterior face area
Very anterior face area
Anterior tip MFS
Anterior tip MFS
Posterior
RH
LH
Sub-19
Human:
Faces
Bodies
Hands
Overlap human categories
Characters:
Words
Fake script
Numbers
Overlap characters categories
Overlap human and characters
Objects
Overlap human and objects
Overlap characters and object
Overlap between human, characters and objects
First/posterior hand/word/face area
Second/middle hand/word/face area
Third/anterior hand/word/face area

*Supplementary Figure 2.* Category-selective regions shown upon annotated right (RH) and left (LH) hemisphere ventral surfaces of all our participants, divided over 5 pages. Color legend on the right. Selective activation (contrast of one versus all other categories except fixation, $p < 0.05$, FWE corrected) to faces in orange, to bodies in ochre, to hands in yellow, to words in dark blue, to fake script in medium blue, to numbers in light blue, and to an objects category (chairs) in medium green. Overlap between selectivity for different human-related categories in red, overlap between selectivity for different character categories in purple, overlap between selectivity for human-related categories overlapping with character categories in brown, overlap between selectivity for human-related categories overlapping with selectivity for the objects category in light green, overlap between selectivity for character categories overlapping with the objects category in dark green, and overlap between selectivity for human-related, characters and the objects category in pink. Annotations were made using a circle, each linked to a square, with the number 1, 2 or 3 to indicate the first, second or third hand (yellow), word (dark blue), and face (orange) area. They also include several arrows that point to the posterior objects region (in green), the pFus word area (dark blue), the anterior tip of the mid-fusiform sulcus (white), a possible third body instead of the third hand area (ochre) and lastly, arrows pointing to even more anterior areas (color depends on the category). All arrows are accompanied by clarifying text.

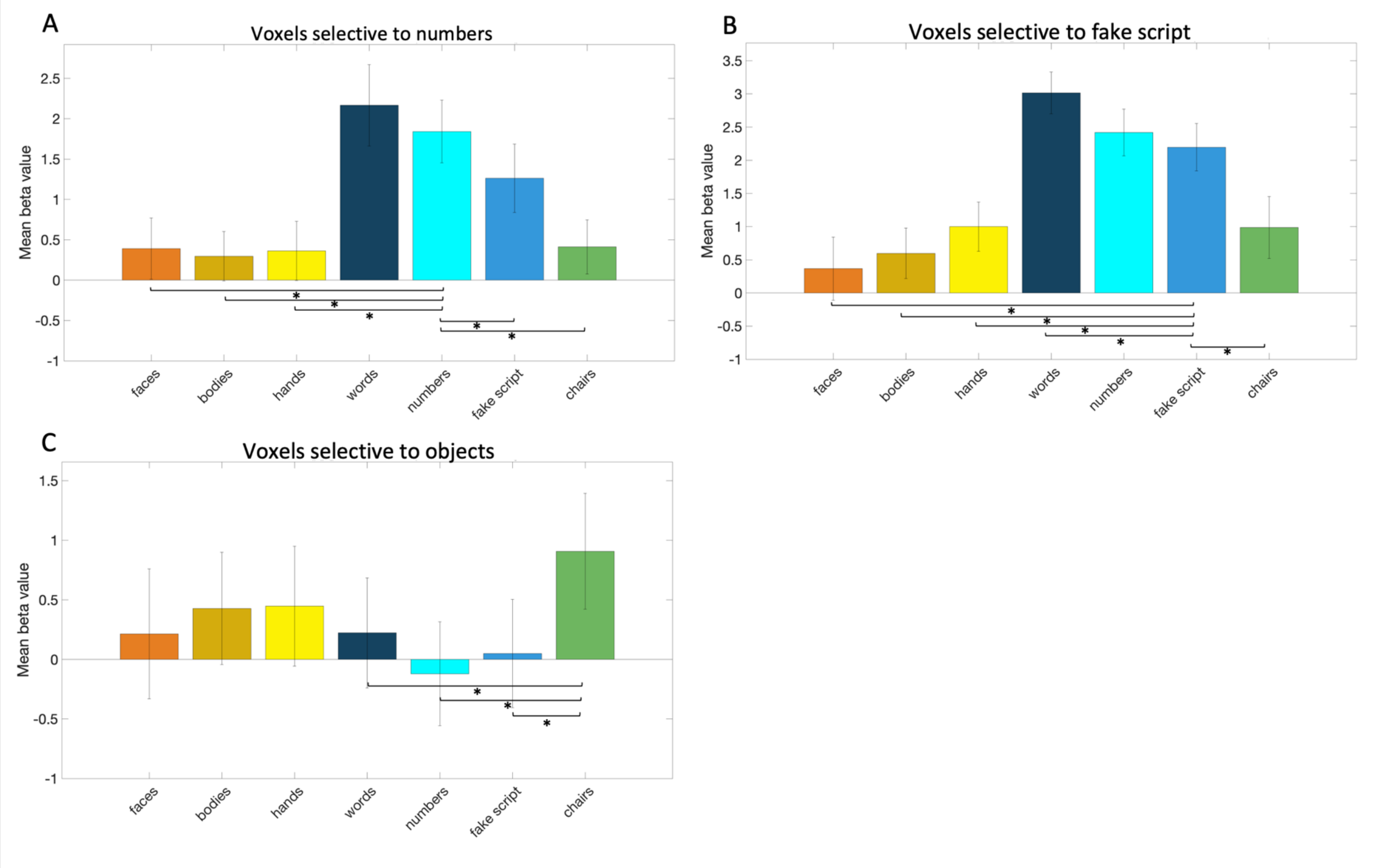


*Supplementary Figure 3.* The response in number- (A), fake script- (B) and object- (chair) (C) selective voxels of the left hemisphere to seven categories (faces, bodies, hands, words, numbers, fake script and objects: chairs). Responses were calculated using data that were independent from the data used to select the voxels (see Methods). Error bars represent the standard error, and the lines and stars indicate which paired t-tests between conditions were significant ($p$ < .008).

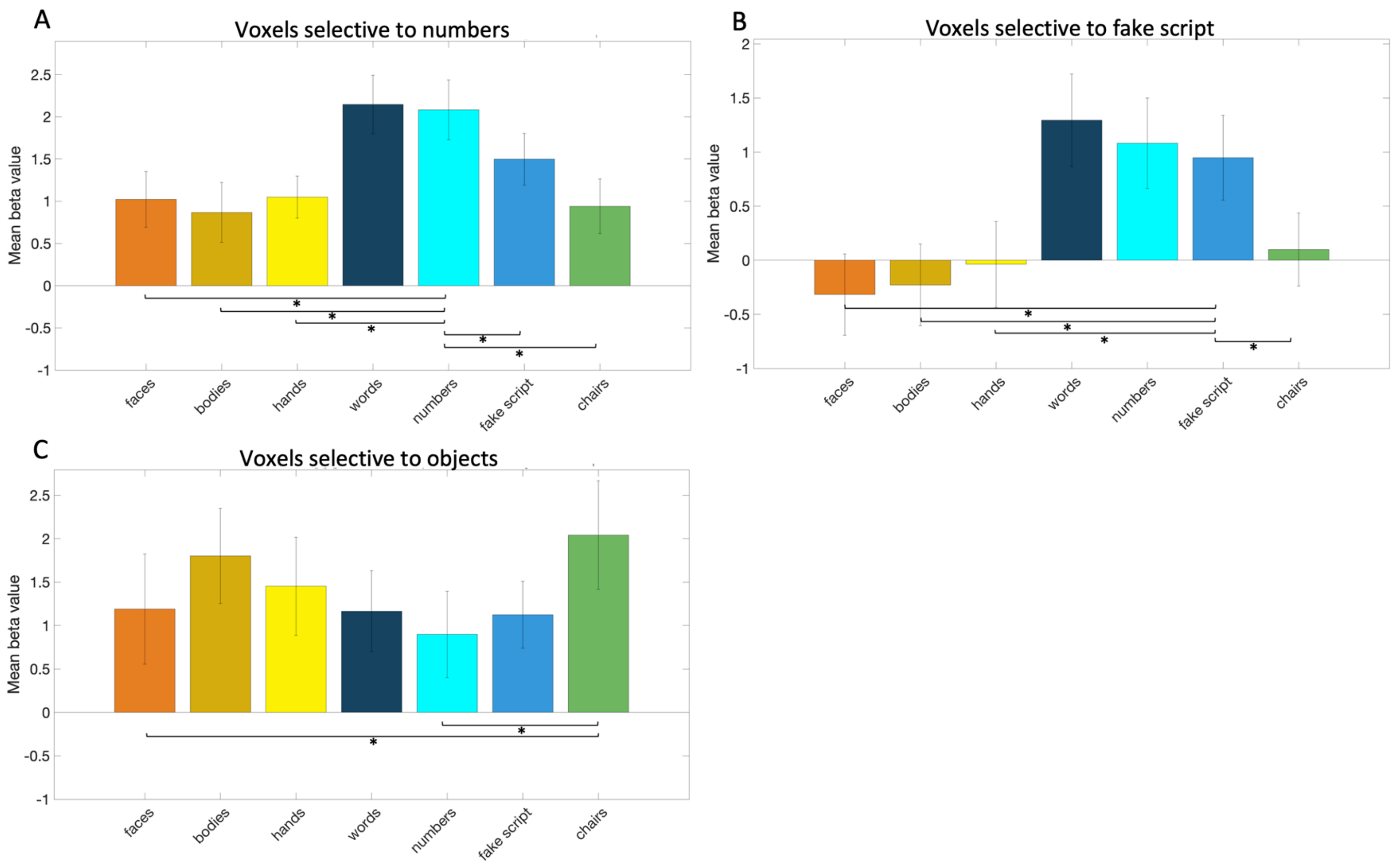


*Supplementary Figure 4.* The response in number- (A), fake script- (B) and object- (chair) (C) selective voxels of the right hemisphere to seven categories (faces, bodies, hands, words, numbers, fake script and objects: chairs). Responses were calculated using data that were independent from the data used to select the voxels (see Methods). Error bars represent the standard error, and the lines and stars indicate which paired t-tests between conditions were significant ($p < .008$).

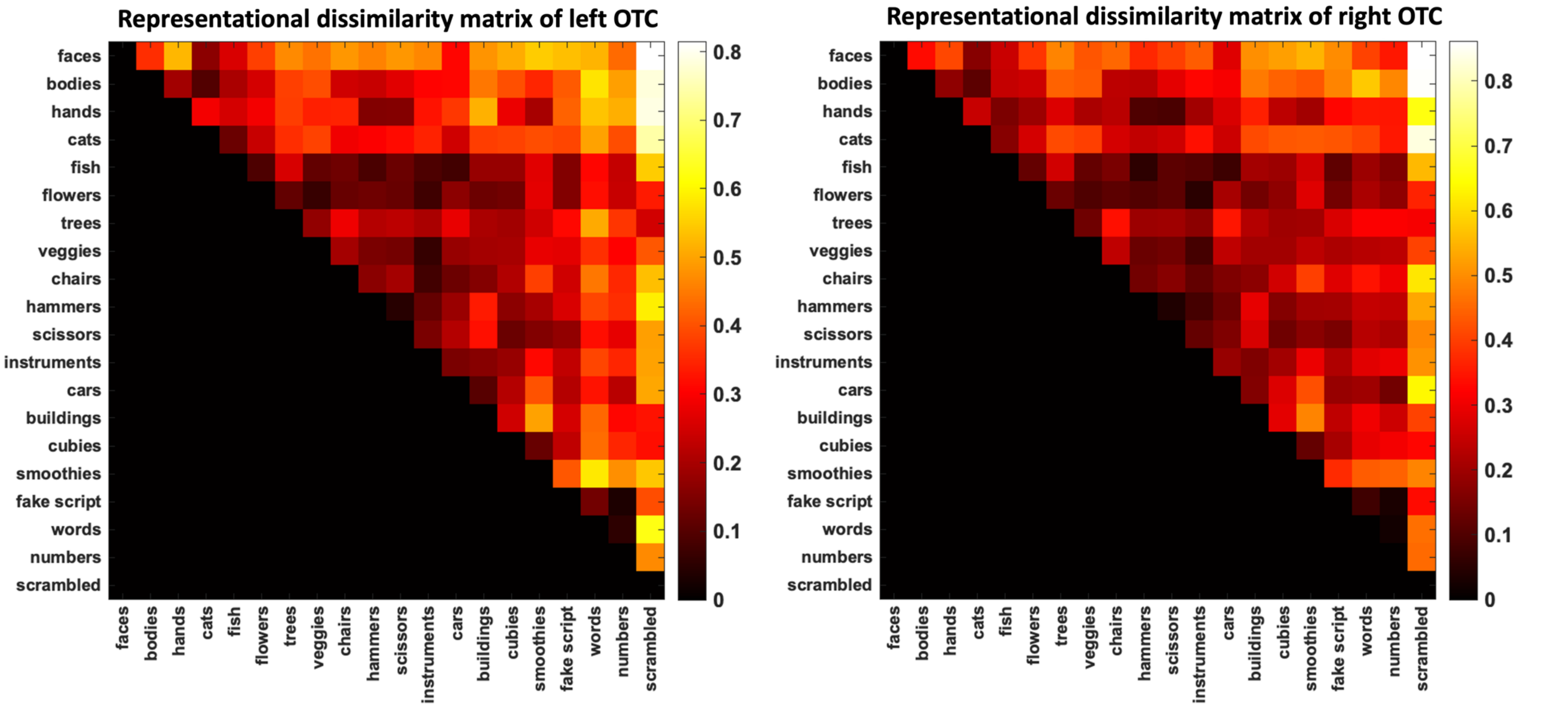


*Supplementary Figure 5.* The representational dissimilarity matrix of the left OTC (left panel) and right OTC ROI (right panel). These matrices were obtained by averaging across the left or right OTC normalized matrix from each participant. The labels on the rows/columns describe the category pair and the color of a cell in the matrix represents the averaged normalized LDC value, where dark red signifies a small distance between the neural representations of the two categories, whereas yellow signifies a large distance.

**Supplementary Results**

**3.1 Functional neuroanatomy of ventral OTC**

Based on the observed consistent relative position of the three types of category-selectivity (words, faces, hands), we defined, as described in the text in section 3.1.2.1, a first hand cluster with activity in the lateral occipital sulcus and inferior occipital sulcus. More medial to that, we defined the IOG face cluster, and around this cluster the first word cluster (all within the inferior occipital gyrus). The second hand cluster was defined along the middle fusiform gyrus and occipitotemporal sulcus. We decided to define word activity in between these two hand areas as the second word area, which lied more lateral on the fusiform gyrus/occipitotemporal sulcus than the face area pFus/FFA1. We also defined here a pFus word area when there was word activity present within the pFus face area and/or more medial to that face area. We also located the mid-fusiform sulcus which can help locate pFus/FFA1 and mFus/FFA2, according to Weiner and colleagues (2014). Lastly, below the second hand area (and in between the third hand area if present in anterior fusiform gyrus), we defined mFus and word activity around mFus as the third word area.

Sometimes participants showed some variability from this standard pattern. In these cases, we used this standard pattern as much as possible to make decisions about which activity to include into areas, but nonetheless these decisions are somewhat uncertain and subjective. This variability was the most obvious when certain areas were missing and this type of variability was made explicit in the above results sections by mentioning which subjects digressed from the standard (and this is also summarized in Supplementary Figure 1). Some other examples can also be described. For example, in the right hemisphere of subject 12, there was a part of the third word patch that in principle could be defined, according to the standard pattern, as the second word patch as it was located more posterior than the second hand area, but given it was located far away of the other part of the second word area and much more medial, we believed this patch fitted better as a part of the third word area. These kinds of decisions had to be made more often in the right hemisphere where the relative pattern of areas was much less consistent, as described in detail in the supplementary results below. As an example of the left hemisphere, in subject 8, the most anterior patch of the second word area might be a part of the third word area, as it is located more anterior than the second hand area (following the standard pattern). Here, we decided however to include this patch as part of the second word area because it was located on the same part of the sulcus.

As a last note, we emphasize that the squares on Figure 4 and Supplementary Figure 2 are not perfect: it was not always possible to put a square around all activity that could be included into, for example, the third word area because this would mean a chaotic visualization using either many squares or irregular polygons that would hide too much of the activity on the surface. Take, for example, subject 11: a small word selectivity patch was located medial to the second hand area and third face area and could also be defined as (part of) the third word cluster. We did not put another square around this patch as it was small and we attempted to keep the surface annotations clean by annotating sparingly. The same was true for, for example, subject 12: there was a patch of word selectivity between the second and third face area, that was in our opinion a part of the second word patch (as it was located more posterior than the second hand and third face area), but we did not want to put yet another square around this, so that the visualization of this consistent pattern of areas would still be clear from a quick glance at the surface.

**3.1.3.1 The location of hand selectivity cannot serve as a reference point to locate word and face selectivity in the right hemisphere**

Then, we compared the locations of these areas in the right hemisphere to the locations in the left hemisphere and investigated if the hand areas in this hemisphere could also consistently locate the VWFA subareas and the typical face areas, like in the left hemisphere. In conclusion, the hand areas could not serve as a reference landmark like in the left hemisphere, in part due to the more complex nature of the hand and word selectivity in the right hemisphere. For more details, see below.

First, we investigated the posterior part along the inferior occipital gyrus of the right ventral surface of the OTC, first investigating the word selectivity. In all subjects, we found a cluster of selectivity to some type of characters here. Compared to the left hemisphere, the selectivity (whether it was to words or only to other characters) was often smaller, more distributed and in some subjects, none (or only minimal) of this selectivity was located very close to the posterior face area (IOG), as it did in the left hemisphere. Second, regarding the face selectivity (IOG), the differences between the left and the right hemisphere were minor: it appeared in the usual location and size. Third, regarding the hand selectivity, the location of the posterior hand area differed from the left hemisphere in several subjects. In subject 1, 3, 7, 10, 11 and 18, the location was more anterior (including the fusiform gyrus), and this made it difficult (in subject 7 especially) to discern it from the middle hand area as we found in the left hemisphere. Lastly, in the left hemisphere in some subjects, there was a small cluster of selectivity to objects (or some kind of overlap in selectivity for objects and character or human-related categories) in between the posterior hand cluster and the posterior word and face cluster. We also found this in 10 subjects in the right hemisphere (subject 1, 2, 6, 7, 9, 10, 11, 12, 13, 15), around the posterior word and face cluster, but not always exactly between those two areas and the posterior hand cluster (presumably due to the more complex location of this hand area in this right hemisphere).

Next, we investigated the middle fusiform gyrus on the ventral surface of the right OTC, first investigating the word selectivity. The second/middle cluster of selectivity to words was more unclear to define than the posterior one. This second word selectivity cluster appeared consistently lateral (on the occipitotemporal sulcus or lateral fusiform gyrus) in the left hemisphere and on the side of the second face cluster (pFus), but this seemed to vary in the right hemisphere. In subject 1, 2, 3, 5, 8, 14 and 15 this second word cluster was located more medial than expected on the fusiform gyrus, sometimes accompanied by another small cluster that was more lateral (subject 2, 8). Second, regarding the second cluster of selectivity to faces (pFus): this area was harder to define in the right than in the left hemisphere, because it was often less prominent. A possible reason could be the due to the more medial presence of the second word cluster like just discussed. Third, regarding the hand selectivity, we found the second hand region in many subjects, but it was harder to define in the right hemisphere compared to the left, due to the first hand area sometimes appearing in an unexpected location (see above). Lastly, in the left hemisphere in some subjects, pFus was joined, or broken up into two, by a cluster of selectivity to words and/or other characters. This pFus word area was more difficult to discern in the right hemisphere due to pFus being absent more often or being less prominent. In addition, the often more medial location of the second word cluster also made this difficult to do. We could only find possible candidates for this definition in subject 2 and 16 but these clusters consisted only of selectivity to the numbers category and not to words.

Lastly, we investigated the anterior fusiform gyrus of the right ventral surface of the OTC. We often found face selectivity here, but in contrast to the left hemisphere, we rarely found word or hand selectivity. In the left hemisphere we noted in some subjects an even more anterior cluster of selectivity to faces, and sometimes words. Similarly in the right hemisphere, subject 1 (although it might fit as a part of mFus too), 4, 6, 9, 12, 14 and 19 showed a cluster of selectivity to faces here.

Subject 3 showed selectivity to both faces and bodies. Subject 5 showed selectivity to both faces and words. Subject 16 showed selectivity to numbers, subject 17 to words and subject 18 to hands.

We would like to conclude that for the organization in the right hemisphere, the hand areas could be less of a guide to locate the other types of selectivity, because they were in another (less consistent) location compared to the left hemisphere, or small (sometimes so small that it was unclear if the area was present), or the hand cluster was just not present at all (in case of the middle hand area for some subjects). In addition, the presence and location of the word clusters was also more complicated than in the left hemisphere.
